# When Proteins Go MAD—Misfolded, Amplified, Detected: Advances in α-Synuclein Pathophysiology and RT-QuIC Detection

**DOI:** 10.1007/s12035-025-05600-2

**Published:** 2026-01-09

**Authors:** Naďa Labajová, Adam Polák, Ondrej Cehlár, Pavle Križan, Jozef Hritz, Martin Kolisek, Matej Škorvánek, Rostislav Škrabana, Branislav Kovačech, Norbert Žilka

**Affiliations:** 1 Institute of Neuroimmunology, Slovak Academy of Sciences, Bratislava, Slovakia; 2 National Centre for Biomolecular Research (NCBR), Faculty of Science, Masaryk University, Brno, Czech Republic; 3 Central European Institute of Technology (CEITEC), Masaryk University, Brno, Czech Republic; 4 Jessenius Faculty of Medicine in Martin, Biomedical Centre Martin, Comenius University in Bratislava, Martin, Slovakia; 5 Department of Neurology, Faculty of Medicine, P.J. Safarik University, Kosice, Slovakia; 6 Department of Clinical Neurosciences, Faculty of Medicine, P.J. Safarik University, Kosice, Slovakia; 7 Department of Neurology, University Hospital L. Pasteur, Kosice, Slovakia

**Keywords:** α-Synuclein, RT-QuIC, Parkinson’s disease, Aggregation, Neurodegeneration, Synucleinopathies

## Abstract

Α-Synuclein (α-Syn) aggregation and fibrillation are pathological hallmarks of several neurodegenerative disorders, collectively termed synucleinopathies. The misfolded α-Syn protein exhibits a prion-like seeding behavior, promoting misfolding, intracellular spread, and progressive neurodegeneration. Recent advances in structural biology have revealed critical insights into the conformational heterogeneity of α-Syn aggregates and their strain-specific properties across distinct synucleinopathies. In parallel, significant progress has been made in biomarker development, particularly with the arrival of seed amplification assays. Among these, Real-Time Quaking-Induced Conversion (RT-QuIC) has emerged as a highly sensitive, specific, and scalable method for detecting pathogenic α-Syn species in cerebrospinal fluid and other tissues. This review summarizes the latest findings from structural studies on α-Syn oligomers and aggregates, their relevance to disease mechanisms, and highlights RT-QuIC as the most clinically advanced and rapidly evolving assay. We discuss its potential for early, biomarker-driven diagnostics, patient stratification, and clinical implementation.

**Supplementary Information:**

The online version contains supplementary material available at 10.1007/s12035-025-05600-2.

## Introduction

Synucleinopathies are neurodegenerative disorders marked by the pathological aggregation of α-synuclein (α-Syn). α-Syn is a small protein that plays a crucial role in synaptic function and neurotransmitter release. Its role is multifaceted, influencing several stages of synaptic vesicle trafficking. Specifically, it is implicated in vesicle clustering, docking, and homeostasis, as well as SNARE complex assembly, which is essential for vesicle fusion and neurotransmitter release [1–8]. α-Syn is not only a protein integral for the process of neurotransmission but also an essential signaling protein in the process of hematopoiesis [9]. α-Syn is present in the nucleus of neurons [10], in mitochondria [11], Golgi apparatus [12], endoplasmic reticulum [13], and the endolysosomal system [14]. Under certain conditions, such as genetic mutations, environmental factors, or homeostasis defects, α-Syn can misfold and aggregate into toxic forms, leading to neurodegeneration.

Under pathological conditions, misfolding of α-Syn leads to the formation of β-sheet-rich oligomeric and fibrillar aggregates (amyloid fibrils), forming inclusions that are resistant to degradation [15]. Misfolded α-Syn can propagate in a prion-like fashion, driving neuronal death and neuroinflammation [19]. This process contributes to the progressive decline in motor and cognitive functions. Synucleinopathies include Parkinson’s disease (PD), dementia with Lewy bodies (DLB), and multiple system atrophy (MSA). As the population ages, the prevalence of synucleinopathies is increasing, underscoring the need for accurate, early diagnostic methods.

**Fig. 1:**
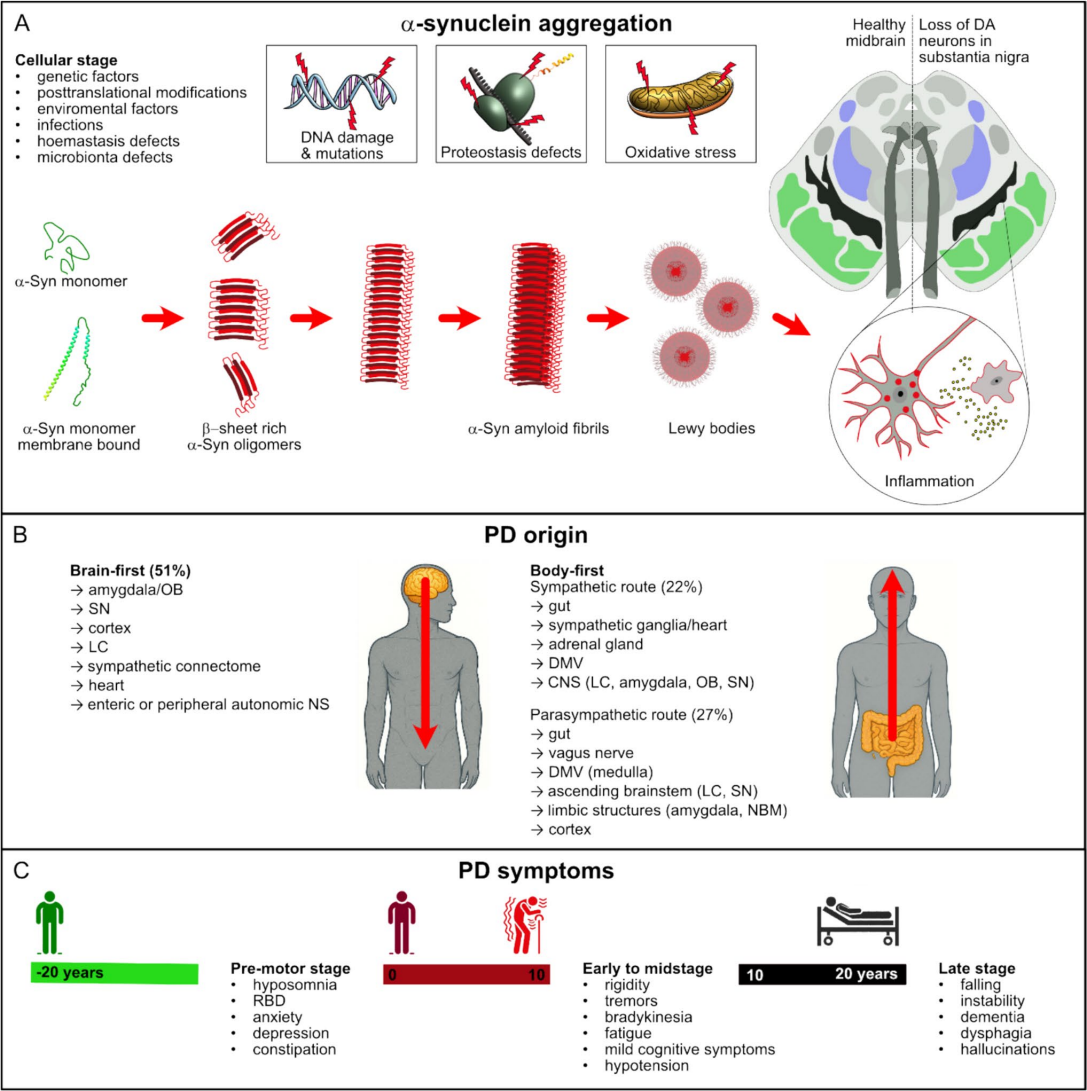
Schematic representation of Parkinson’s disease origin and progression. The illustration highlights the timeline of pathological events in Parkinson’s disease (PD). **A** The neurodegeneration begins years before the onset of clinical motor symptoms at the cellular level and is likely driven by a multifactorial interplay of pathogenic mechanisms. **B** The α-Syn pathology may originate and spread from the enteric, renal, or peripheral autonomic nervous system (the body-first type) or from the olfactory peduncle (the brain-first type). Brain-first type exhibits little to no peripheral pathology during the pre-motor stage, whereas body-first cases show pronounced early autonomic involvement. **C** Over the course of several years, PD progresses through distinct yet overlapping stages, each marked by the most common non-motor and motor symptoms. Abbreviations in the illustration: DA, dopamine; OB, olfactory bulb; SN, substantia nigra; LC, locus coeruleus; NS, nervous system; DMV, dorsal motor nucleus; CNS, central nervous system; NBM, nucleus basalis of Meynert; RBD, rapid eye movement sleep behavior disorder

PD is the most prevalent synucleinopathy and the second most prevalent neurodegenerative disorder after Alzheimer’s disease (AD) [20, 21]. Its etiology is multifactorial, involving both genetic and environmental factors. In PD, α-Syn inclusions are found either within neuronal processes, referred to as Lewy neurites, or within neuronal cell bodies, where they are known as Lewy bodies [16]. PD is defined by the progressive loss of dopamine neurons in the substantia nigra. Activation of microglia and release of pro-inflammatory cytokines establish a chronic neuroinflammatory environment that contributes to neuronal degeneration (Fig. 1A).

Recent studies have proposed that Parkinson’s disease progression might follow different trajectories, tentatively described as brain-first and body-first subtypes (Fig. 1B). In brain-first cases, α-Syn aggregation begins within central nervous system structures such as the amygdala or olfactory bulb, with peripheral involvement occurring later. By contrast, body-first cases are characterized by early α-Syn pathology in the autonomic nervous system, including the vagus nerve or sympathetic ganglia, leading to pronounced non-motor symptoms before the onset of classical motor features [22–24]. Defining brain-first and body-first subtypes and mapping their differential involvement of central versus peripheral tissues is critical for understanding PD progression and developing tailored early diagnostic strategies.

The clinical course of PD may progress from the prodromal to the advanced stage over the course of several decades. Stages of PD and most common symptoms include the pre-motor stage, characterized by non-motor features such as constipation, hyposmia, and REM sleep behavior disorder (RBD). Early disease presents with cardinal motor symptoms, including tremor, rigidity, and bradykinesia. The motor impairment progresses, accompanied by postural instability, gait disturbances, and fluctuations in treatment response. The late stage is defined by severe motor disability, dementia, hallucinations, and pronounced autonomic dysfunction. PD is usually diagnosed in later stages after the manifestation of motor symptoms, including bradykinesia (slowness of movement), resting tremor, and rigidity (Fig. 1C).

Genetic background can also modulate disease progression, and young-onset cases often have a genetic background. Nevertheless, the age of onset and disease phenotype may vary widely depending on the underlying genetic variant. Specific genetic backgrounds, such as multiplications of the SNCA gene (coding α-Syn) or different mutant variants of LRRK2 (coding Leucine-rich repeat kinase 2), PRKN (coding Parkin, E3 ubiquitin ligase), or GBA (coding β-Glucocerebrosidase) genes, are typically associated with distinct trajectories of motor decline, cognitive involvement, and non-motor symptom burden [25].

When neurodegeneration spreads beyond the nigrostriatal system, cognitive functions are also impaired. Up to 80% of PD patients develop dementia (Parkinson’s disease dementia, PDD), making PDD the second most common neurodegenerative dementia [26, 27]. PDD emerges in PD patients years after motor symptom onset and affects mostly executive functions, attention, visuospatial functions, and memory.

In contrast to PDD, DLB presents with cognitive symptoms that precede or coincide with motor problems. DLB is the third most common neurodegenerative dementia, after AD and PDD. Similar symptoms to AD often lead to its misdiagnosis [28, 29]. However, its distinct α-Syn pathology can aid differential diagnosis. MSA, a rare but aggressive neurodegenerative disease, disrupts autonomic and motor functions. It manifests in two main subtypes: Parkinsonian variant (MSA-P), which mimics Parkinson’s disease with rigidity and bradykinesia, and cerebellar variant (MSA-C), characterized by ataxia and gait disturbances [30]. In MSA, α-Syn aggregates predominantly form glial cytoplasmic inclusions, also known as Papp–Lantos bodies, which are structures located in the cytoplasm of oligodendrocytes [17, 18]. Although neuronal cytoplasmic inclusions can also be present in MSA, they display a distinct regional distribution compared to Lewy bodies, especially in the putamen, pontine nuclei, and inferior olivary nuclei [18].

The prevalence and incidence of synucleinopathies, which are affecting millions of people worldwide, are anticipated to rise significantly in the coming decades, primarily due to global demographic shifts leading to an aging population. For instance, the prevalence of PD is expected to increase by 20% by 2030 [31]. This trend underscores the urgent need for enhanced diagnostic methods, preventive strategies, and therapeutic interventions to address the growing social, healthcare, and economic burden that these neurodegenerative disorders inflict.

This review provides an integrated overview of the structural and diagnostic dimensions of α-Syn research. We first examine the conformational diversity and aggregation pathways of α-Syn, from monomers to oligomers and fibrils, to clarify their biochemical and pathogenic significance. We then summarize current diagnostic applications of seeding amplification assays (SAAs), particularly recent advances in Real-Time Quaking-Induced Conversion (RT-QuIC), highlighting technological progress, clinical relevance, and future directions for standardized detection of synucleinopathies. The “MAD” framework of this review—Misfolded, Amplified, Detected—mirrors the path of α-Syn from molecular misfolding and aggregation, through amplification in seeding assays, to its detection by RT-QuIC and related diagnostic approaches.

## MAD—Misfolded and Aggregated: α-Synuclein Structure and Pathophysiology

Understanding the peculiarities of α-Syn structure and its implications in physiology and pathophysiology is crucial for identifying biomarkers that differentiate normal α-Syn from its pathological forms. Investigating its pathophysiology, including misfolding, oligomerization, aggregation, and prion-like propagation, enabled the development of highly sensitive seed amplification assays (SAAs) and Real-Time Quaking-Induced Conversion (RT-QuIC) assay. The same nucleation–elongation processes that drive the conversion of monomeric α-Syn into oligomers and fibrils underlie the principles of SAAs and RT-QuIC, in which pathological seeds catalyze the aggregation of recombinant substrates in vitro. Variations in fibril morphology, growth kinetics, and strain-specific structural motifs directly influence the signal profiles of the assay and have the potential to discriminate between different synucleinopathies.

### α-Synuclein Structure

α-Syn is a small, 14 kDa protein, composed of 140 amino acids encoded by the SNCA gene, and consists of three main domains: N-terminal, central hydrophobic, and C-terminal. The N-terminal domain has a propensity to form an amphipathic α-helix that facilitates membrane binding, particularly to synaptic vesicles [32]. In addition, the N-terminal and hydrophobic region contain seven 11-mer imperfect repeats, which are involved in α-Syn filament formation [33, 34]. Especially, the central hydrophobic domain is highly aggregation-prone, playing a key role in fibril formation [35–38]. The C-terminal negatively-charged acidic domain may interact with Ca^2+^ ions and exhibits chaperone-like activity [35, 38, 39].

Over the past three decades, it has become clear that the native physiological state of α-Syn is complex. Protein nuclear magnetic resonance spectroscopy (NMR) and molecular dynamics simulations showed that α-Syn mostly populates conformations whose radius of gyration and solvent accessible surface area are between 20 and 50% greater than those of compact globular states [40]. The peculiar conformational character of α-Syn can be described as intrinsically disordered, with a strong tendency to fold at specific parts of its molecule [41, 42].

**Fig. 2:**
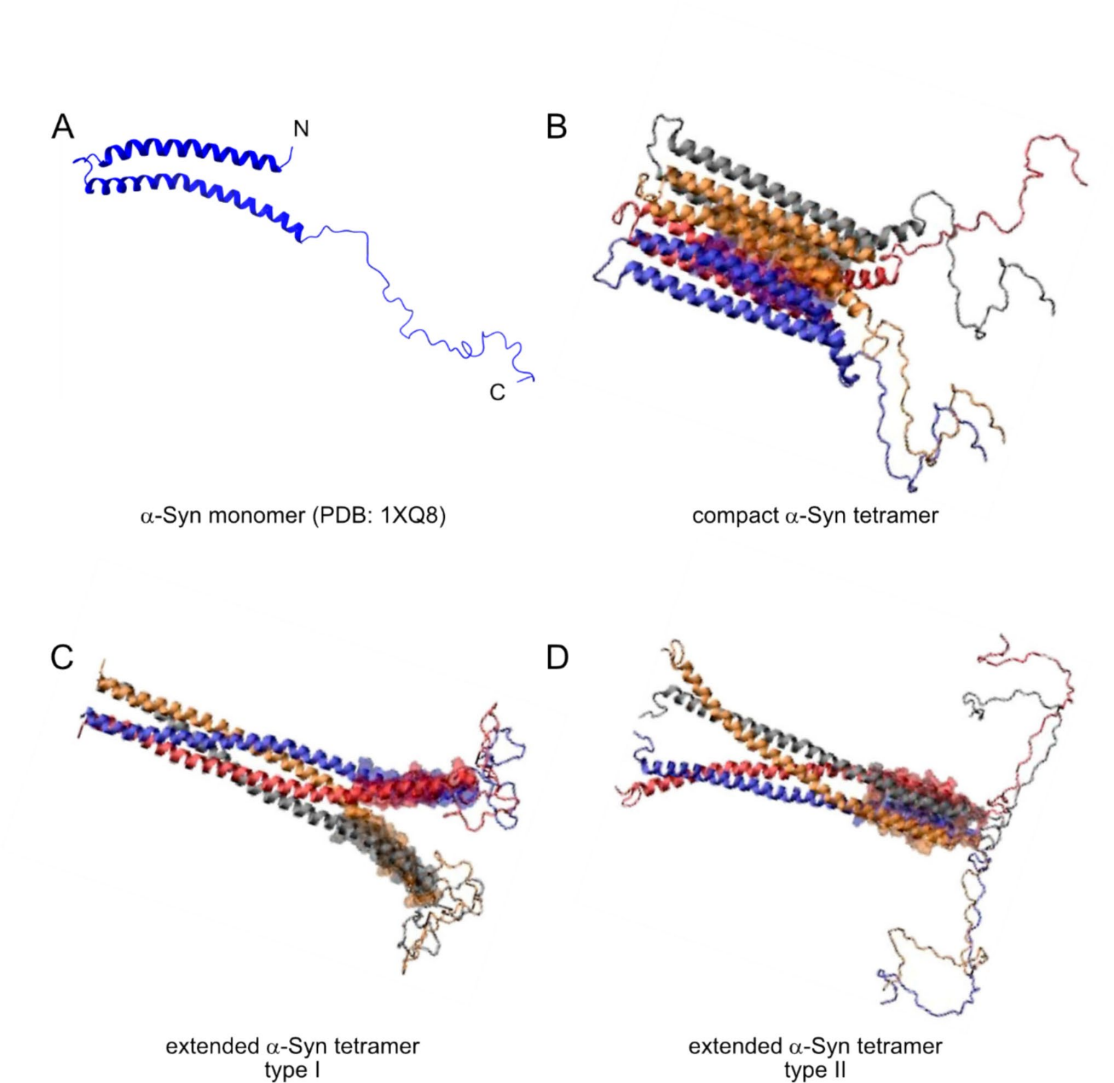
The structures of the helical α-Syn monomer and tetramer. In the soluble state, α-Syn lacks a stable tertiary structure and is considered an intrinsically disordered protein. **A** Upon binding to cellular membranes or negatively charged micelles, α-Syn adopts a curved α-helical conformation (PDB structure 1XQ8). α-Syn can also form a helical tetramer, which, depending on the environment, can be compact (**B**), with inward-pointing helices creating a hydrophobic core, or extended (**C**, **D**), consisting of coiled-coil structures. Tetramer conformations are adapted from Bhattacharya et al. (2024) published under CC BY 4.0 license

Disordered α-Syn can form a helical structure when associated with cell membranes or other negatively charged micelles [43]. This form of bound α-Syn (Fig. 2A) forms two anti-parallel helices that span from V3-V37 and K45-T92 residues connected by a well-ordered linker. The second helix is followed by a short, ordered extension spanning from G93-K97, and the rest of the sequence (D98-A140) is disordered in the solution. The helices are curved, which allows for more intimate interaction of α-Syn and the round surfaces [43].

Later, it was suggested that helical α-Syn can assemble into a tetramer in many human cells, including erythrocytes, and that the tetramers can form independently of charged surfaces [44]. A structure of compact (Fig. 2B) or extended (Fig. 2C, D) tetramers has been postulated based on NMR, CryoEM, and modeling data [45–47]. The compact tetramer is formed via hydrophobic packing of the central regions [46, 48]. In the proposed extended tetramers, coiled coil structures are assembled [45]. The compact helical tetramer conformation, which most probably prevails in the cytosol, could interact with highly charged and neutral lipid bilayer membranes, retaining its compact conformation, but could also shift to an extended conformation on moderately charged membranes. Thus, the dynamic equilibrium of helical tetramers may vary in different cellular environments depending on the membrane lipid composition [45, 46].

Whereas the recombinant disordered monomeric α-Syn can be obtained relatively easily, preparation of the helical tetramer is more challenging. Over the past decade, the process has been optimized, relying on *in-cell* N-acetylation and gentle purification steps to maintain the tetramer stability [41, 49].

The consensus right now is that α-Syn exists in dynamic equilibrium between the monomeric and tetrameric states. It has been proven that mutations in the imperfect repetitive regions of KTKEGV cause α-Syn to lose its helical propensity and favor the monomeric state [50]. Neurons treated with the mutated and more toxic α-Syn that cannot form tetramers were shown to lose their viability [51]. This is highly relevant for understanding the pathogenesis of synucleinopathies, as one of the most common disease-associated mutations, E46K, is the type of mutation that disrupts the KTKEGV repeats [52].

Interestingly, the tetramer is less prone to aggregation in vitro, where even after a 2-week incubation period, the samples did not yield fibrils [44, 53]. Monitoring the tetrameric form of α-Syn may thus have diagnostic potential. It was found that PD patients had lower levels of tetrameric α-Syn in their blood samples, and that asymptomatic G51D carriers and G51D patients with PD also had reduced tetrameric α-Syn. However, further development of the diagnostic assay depends on the development of specific antibodies against tetrameric α-Syn, which could be used in an ELISA format [54].

### α-Synuclein Oligomers

Oligomeric forms of α-Syn are considered to be the most neurotoxic species in synucleinopathies [55]. α-Syn oligomers can be composed of varying numbers of monomers, but they usually include from 11 to around 40 monomers [56]. Single-molecule Förster resonance energy transfer (FRET) studies have shown that α-Syn transitions through two distinct oligomeric states: a loosely packed, protease-sensitive, benign type A and compact, more protease-resistant, toxic type B. Type B oligomers adopt a β-sheet-rich structure with exposed hydrophobic surfaces, which distinguishes them structurally and functionally from the disordered, extended type A oligomers [57].

**Fig. 3:**
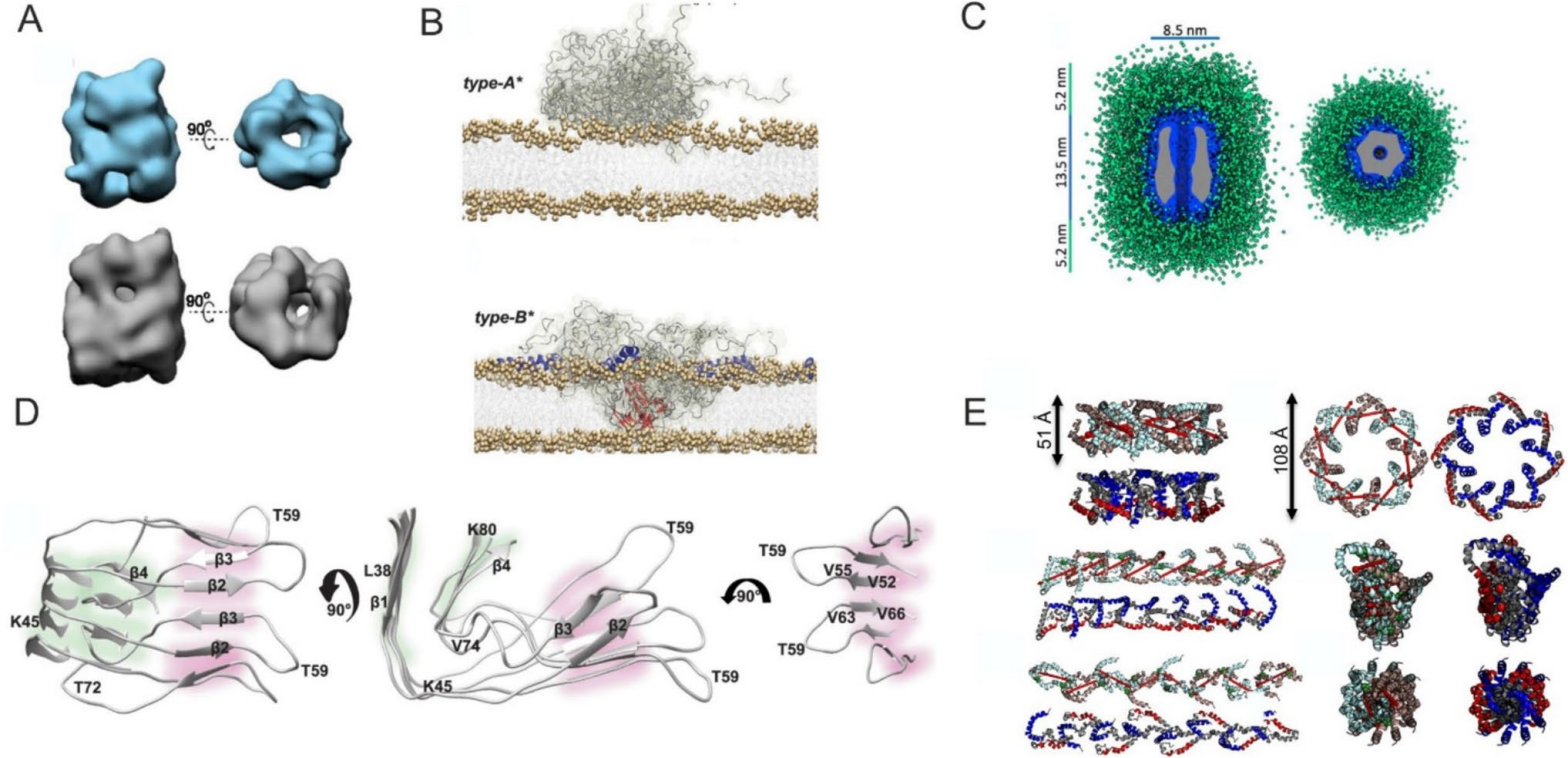
Structural models of α-Syn oligomers. **A** 3D reconstruction of the average structure for the 10S α-Syn oligomer subgroup (blue) and 15S oligomer subgroup (grey). Adapted from [56]. **B** Binding of type A* and type B* oligomers with membranes, where type B* oligomers insert their rigid β-sheet-rich regions into the lipid bilayers and therefore disrupt their integrity. Adapted from [58] with permission from the American Association for the Advancement of Science. **C** SAXS-based model of α-Syn oligomers showing the compact core in blue surrounded by a disordered fuzzy coat shown in green. The Cryo-EM density map is shown inside the oligomer core (gray). Adapted from [60] published under CC BY 4.0 license. **D** Tetrameric intermediate with parallel in register β-sheets highlighted in green background and antiparallel β-sheets in pink background. Adapted from [62] published under CC BY 4.0 license. **E** Circular (top row) and linear/helical/extended (bottom two rows) oligomeric structures derived by the propagation of two dimer models. Adapted from [63] published under CC BY 4.0 license

In addition, structurally related but kinetically trapped oligomers, type B*, were characterized (Fig. 3A). They range from small to large oligomeric states and were divided into two subgroups, 10S and 15S, respectively. The 10S oligomer subgroup usually contained 11–25 monomers, with an average of 18 molecules and an average molecular weight of 260 kDa, and the 15S subgroup contained 19–39 monomers, with an average of 29 molecules and an average molecular weight of 420 kDa. They exhibited features similar to toxic type B, including antiparallel β-sheet structures [56]. Type B* oligomers differ significantly in their capability to bind to and disrupt lipid bilayers with type A* oligomers [58]. While type B* oligomers insert into the hydrophobic core of lipid bilayers, type A* oligomers bind only superficially to the membrane surface (Fig. 3B). The N-terminal 25 residues of α-Syn in type B* oligomers adopt amphipathic α-helical conformations upon membrane binding, which enables deep bilayer penetration and membrane disruption. Type B* oligomers are also more structured. The structured regions span residues 70–88 within the α-Syn hydrophobic region and form a rigid core rich in β-sheets, which is negligible in the type A* oligomers. Type A* oligomers display flexible, poorly ordered structures in the spanning residues 3–36. However, both type A* and type B* oligomers comprise a large number of mobile residues, 45 in type A* and 67 in type B*. Type B* oligomers prepared from α-Syn mutants A30P and (Δ2–9) suppress membrane insertion and reduce the toxicity of type B* oligomers, validating the structural–functional relevance of this region [58]. Van Diggelen and colleagues attempted to provide a classification of α-Syn oligomers prepared under varying conditions in vitro*.* The study revealed a wide range of morphologies, including spherical, annular, cylindrical, prolate, doughnut-shaped, elongated, wreath-like, spheroidal, globular, and ring-shaped [59].

Recently, Santos and coworkers identified two regions in the N-terminal domain between (P1: residues 36–42, and P2: residues 45–57), which are essential for the oligomer-to-fibril transition and can be targeted by short peptides for oligomer disassembly [60]. They tested the peptides on oligomers forming compact, sixfold symmetric super-ellipsoidal structures with a central hollow core and an outer disordered shell (Fig. 3C). The outer shell is formed predominantly by the C-terminus and the first 20 N-terminal residues, while the 36–60 segment adopts a partially folded conformation involved in fibril transition [60]. They also showed that mutation G51D in or P2 region inhibits fibril formation and leads to the accumulation of toxic oligomers. The G51D mutation is clinically relevant, as it leads to an aggressive, early-onset form of PD characterized by symptoms that overlap with both PD and MSA [61].

In the presence of anionic lipid vesicles, a toxic prefibrillar oligomeric α-Syn intermediate (I1) has been isolated along the pathway leading to the formation of fibril polymorph, termed L2 [62]. The oligomer has been characterized as a tetramer containing both parallel, in-register, and antiparallel β-strands. During aggregation, a conversion from an antiparallel β-sheet with a β-hairpin to a parallel in-register β-arc at residue T59 occurs (Fig. 3D). In addition, models of SDS-micelle-bound α-Syn oligomers have been derived from integrative analyses combining several methods. These studies identified two distinct dimer conformations, each consisting of monomers with three α-helices. Oligomerization of these dimers results in circular and elongated structures that exhibit helical patterns along their longitudinal axes (Fig. 3E) [63]. α-Syn oligomers are also predominantly formed by secondary nucleation on fibril surfaces at physiological pH, even in the absence of lipid membranes [64].

Together, these findings highlight the structural and mechanistic diversity of α-Syn oligomers, which not only serve as key intermediates in fibril formation but also set the basis for the emergence of disease-specific filament architectures observed in synucleinopathies. Detection of oligomers is very challenging and requires a method with extreme sensitivity and specificity, but it offers a promising avenue for early and differential diagnosis of neurodegenerative diseases [65]. It was shown that spherical α-Syn oligomers can be internalized by neuronal cells and can act as seeds for amyloid aggregation [66]. The in vivo spreading of α-Syn oligomers after the injection into the olfactory bulb was also shown [67]. Unlike amyloid fibrils, oligomers exhibit reduced Thioflavin T (ThT) fluorescence in vitro, have a more hydrophobic surface, and lower resistance to proteolysis. These features, when used appropriately, can help diagnose early or intermediate disease stages and contribute to differential diagnosis.

In addition, α-Syn oligomers are promising drug targets for which small molecules, molecular chaperons, or antibodies can be designed. More detailed characterization of toxic and non-toxic oligomer pairs can shed light on the specific determinants of oligomer-induced toxicity. Detailed structural characterization of oligomeric species that are either on-pathway fibril precursors or off-pathway competitors of fibril formation will be needed to complement knowledge of the aggregation mechanism. Equally important is determining how closely in vitro–generated oligomers resemble those isolated from human tissue. This similarity will define which experimental models are most relevant for developing pharmacological interventions and diagnostic assays.

### Aggregated α-Synuclein Filaments from Human Patients

The key mechanism driving the accelerated formation and aggregation of oligomers, as well as their prion-like propagation, is monomer unfolding facilitated through secondary nucleation. α-Syn monomers can transiently bind through their positively charged N-terminus to the negatively charged, flexible C-terminal regions of the fibrils. These intermolecular contacts disrupt intramolecular interactions within the monomer, leading to unfolding and promoting alignment of monomers on the amyloid fibril surface [68]. The formation of amyloid fibrils can be monitored in vitro using fluorometric dyes, such as ThT, and is used in diagnostic assays, such as RT-QuIC.

**Fig. 4:**
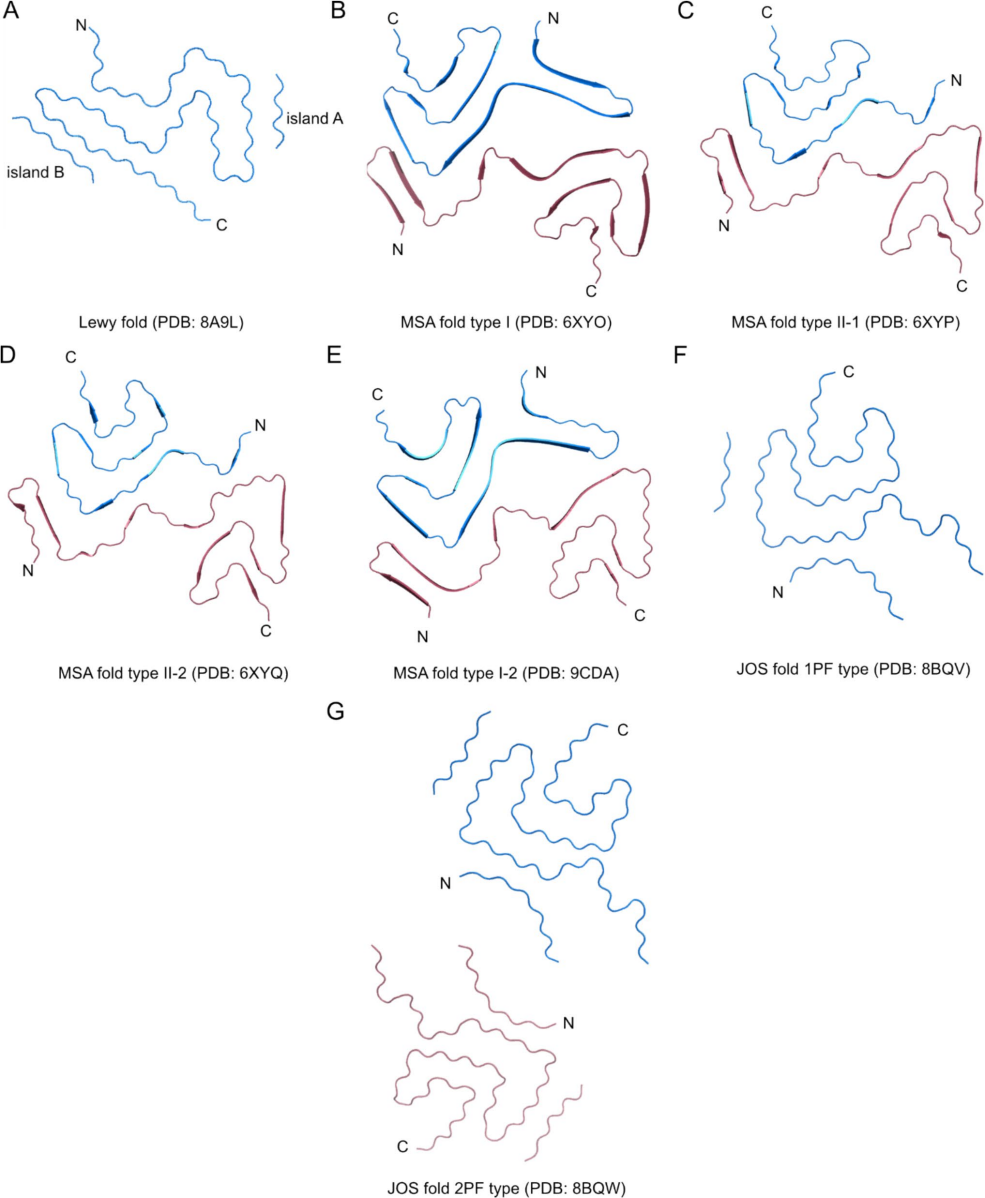
Lewy, MSA, and JOS folds. **A** Lewy fold formed by single-protofilament common to PD, PDD, and DLB, MSA filaments formed by two asymmetric protofilaments units: **A** type I filaments, **C**, **D** type II filaments, **E** subtype of MSA I filaments with mixed features of type I and type II MSA filaments, **F** highly populated (83%) JOS fold filaments formed by a single-protofilament with unidentified protein fragment, **G** less populated (17%) JOS fold filaments formed by two symmetric protofilament units with unidentified protein fragment

The final stage of α-Syn aggregation is amyloid fibrils. Interestingly, fibrils can adopt multiple distinct conformations, even within a single sample—a phenomenon known as fibril polymorphism. They can differ significantly in diameter, helical twist, number of protofilaments, and susceptibility to proteolysis. Substantial differences can also be found between the fibril structures of synucleinopathies, especially between PD and MSA. Yang and coworkers described a single-protofilament structure common to PD, PDD, and DLB termed the “Lewy fold” (PDB: 8A9L; Fig. 4A) from brain samples of patients. The amyloid core involves residues G31–L100, arranged into a three-layered β-sheet structure with nine β-strands (β1–β9) and a right-handed twist. The fold features two additional densities, “island A” and “island B,” packed against β5 and β9, respectively. Residues K32, K34, Y39, K43, and K45 form a cavity that could accommodate a cofactor. In the single-protofilament fibrils in PD, PDD, and DLB, the stability of the filaments is mediated by a combination of polar, hydrophobic, and E35-K80 electrostatic interactions involving water molecules inside the filament [69].

In contrast, MSA filaments extracted from human brains were initially described as three different populations termed type I (PDB: 6XYO; Fig. 4B), type II-1 (PDB: 6XYP; Fig. 4C), and type II-2 (PDB: 6XYQ; Fig. 4D). Type I filaments have the outer interface packed against residues A76-K80, while type II filaments include residues V74-A78 in the inner interface and form a smaller cavity between protofilament subunits. Type II filaments are discriminated from type I by possible phosphorylation of T81 of the larger protofilament subunit. An intramolecular salt bridge between E46 and K80 stabilizes all the reported structures of individual protofilaments. The filaments are, on the other hand, stabilized by the packing of the inner, outer, and central layers [70]. Recently, samples from another MSA post-mortem brains revealed new subtype of MSA filaments type I, termed type I-2 (PDB: 9CDA; Fig. 4E). This population has the C-terminal region oriented outwards from the inter-protofilament interface as in the case of type I filaments; however, the inner interface is packed against V74-A78 as described for type II filaments. These structural features suggest that protofilaments in MSA have the capacity to mix and form hybrid or low-abundance chimeric filament populations [71].

A third distinct patient-derived α-Syn filament conformation, termed the JOS fold, has been associated with juvenile-onset synucleinopathy. It involves the insertion of 7 residues (MAAAEKT) after T22. Two filament populations exhibiting this fold have been extracted from brain samples, comprising either a single-protofilament (PDB: 8BQV; Fig. 4F) or two identical protofilament subunits (PDB: 8BQW; Fig. 4G). In addition to the identified central core (36–100), the solved structures also contain island A (presumably N-terminal part of α-Syn), island B (unidentified protein fragment), and a large non-protein cavity at the interface of double-protofilament fibrils. Interestingly, in vitro–assembled filaments of MAAAEKT α-Syn (PDB: 8CEB, 8CE7) did not adopt the JOS fold. Instead, these filaments exhibited structural characteristics more closely resembling those of MSA filaments with E46-K80 electrostatic interaction, polar, and hydrophobic contacts [72].

### Structures of In Vitro Assembled α-Synuclein Filaments

The filaments assembled in vitro from recombinant α-Syn differ from human brain-isolated structures. They differ in the number, arrangement, and interfacial contacts of protofilaments. Recently, Dhavale and colleagues characterized both single- and double-protofilament fibrils amplified using samples from DLB patients (PDB: 8FPT; Suppl. Figure 1A) [73]. Solid-state NMR and cryo-EM data revealed β-strand regions spanning E34 to V95, involving β-sheet stacking with hydrophobic clusters as in “Lewy fold” [69, 73].

When a mix of type I and type II brain-derived MSA filaments was used for seeding of in vitro α-Syn aggregation, it resulted in two distinguishable populations of filaments with MSA fold [70, 74–76]. First population 1A (PDB: 7NCA; Suppl. Figure 1B) and 1B (PDB: 7NCH; Suppl. Figure 1C), being stabilized by E46-K58 salt-bridge, are similar to previously published rod (PDB: 6CU7; Suppl. Figure 1D) and twister polymorphs (PDB: 6CU8; Suppl. Figure 1E), respectively [70, 75, 76]. The key structural difference between 1A and 1B lies in their protofilament interfaces: rod polymorphs are stabilized by residues 47–56, while twister polymorphs involve residues 68–78 [75]. The second class, 2A (PDB: 7NCG; Suppl. Figure 1F) and 2B (PDB: 7NCI; Suppl. Figure 1G), is stabilized by K45-E46 salt bridge, and the structure is similar to previously published 2A and 2B polymorphs, 6SSX (Suppl. Figure 1H) and 6SST (Suppl. Figure 1I), respectively [70, 74, 76]. Interestingly, seeding only with type 2 MSA filaments resulted in exclusively single-protofilament fibrils (PDB: 7NCK; Suppl. Figure 1J) structurally identical to the protofilament of MSA class 2B filaments [70].

**Fig. 5:**
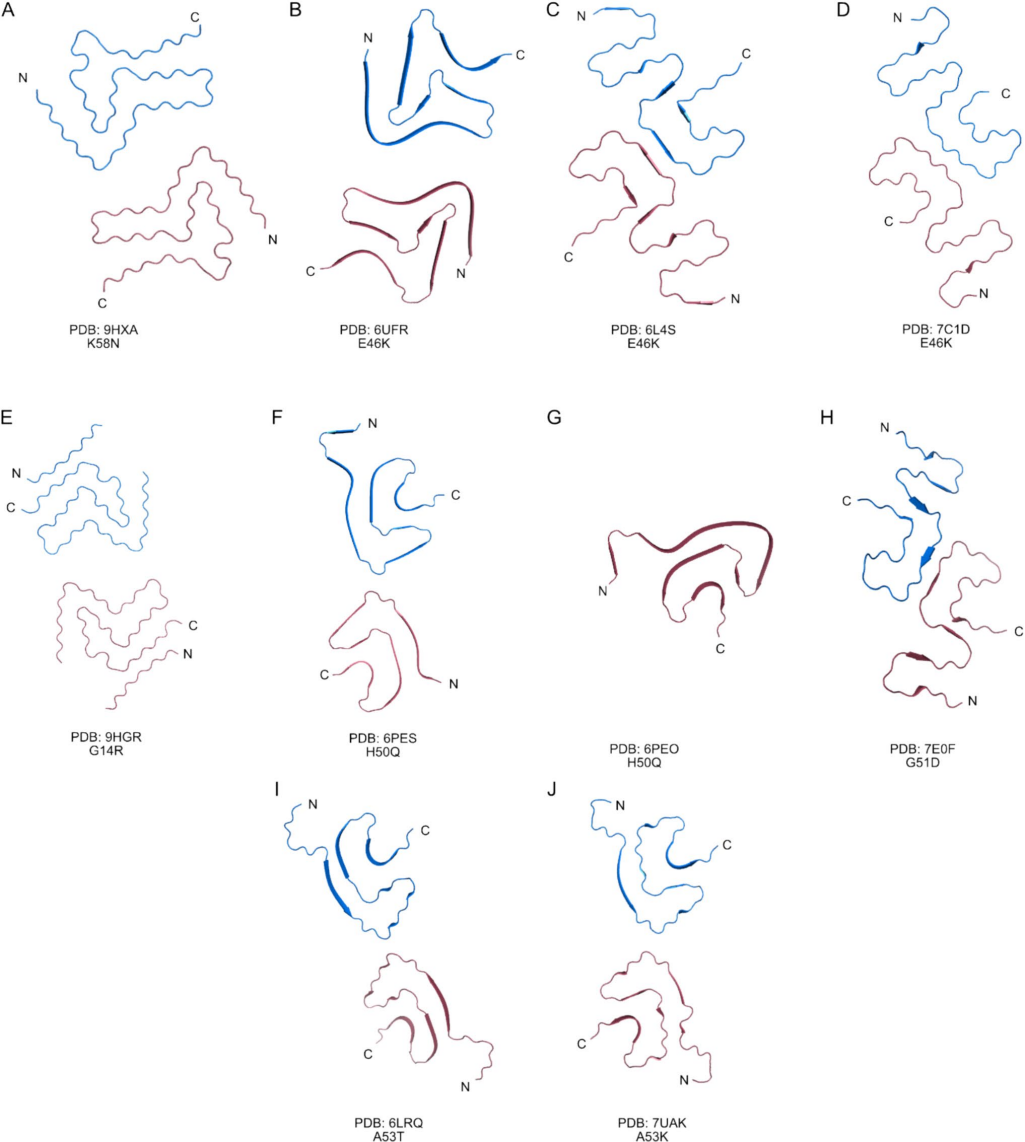
Structures of α-Syn filaments carrying familiar mutations. Recombinant α-Syn fibrils carrying the following familiar mutations: **A** K58N, **B–D** E46K, **E** G14R, **F, G** H50Q, **H** G51D, **I** A53T, and **J** A53E. These mutations are generally associated with early onset and worsen disease progression and pathology by promoting rapid fibril formation, increased cytotoxicity, and the seeding ability of α-Syn

The nomenclature of recombinant α-Syn filaments is rather complicated and previously used Roman numbering to classify fold types, combined with letters (e.g., A and B) to denote variants of the same fold, which was later changed to Arabic numbering by Guerrero-Ferreira and colleagues [74]. Recently, Frey and coworkers further extended the categorization of filaments. They added class 3B and 3C filaments (PDB: 9FYP, 8PIX Suppl. Figure 1K, L), produced at low pH, similar to previously reported structure under PDB ID 6UFR (Fig. 5B) with E46K mutation [77, 78]. They also described type 5 filaments (PDB: 8PK4; Suppl. Figure 1M). But more importantly, they found out that even small changes (in buffer, ionic strength, pH) significantly affect which polymorphs are amplified in vitro. In their cross-seeding experiments, it was the pH, not seed identity, that dictated the resulting polymorph type [78].

The structures of in vitro–formed α-Syn polymorphs are highly relevant for elucidating the molecular events underlying SAAs. These structural insights provide a deeper understanding of how specific conformers of α-Syn seeds propagate and template the conversion of monomeric α-Syn substrates. Consequently, they offer critical information for optimizing assay conditions, improving sensitivity and specificity, and identifying strain-specific signatures that may reflect distinct synucleinopathies. By mapping the structural landscape of α-Syn polymorphs and correlating them with seeding kinetics and ThT fluorescence profiles, researchers can better interpret RT-QuIC readouts and potentially design next-generation diagnostic tools capable of distinguishing between disease subtypes at early or even prodromal stages.

### Disease-Relevant α-Synuclein Mutants and Structures of Mutant Filaments

The influence of familial α-Syn mutations on amyloidogenesis has also been extensively investigated, and several high-resolution filament structures are now available in the Protein Data Bank (PDB). The structures of filaments in the PDB include aggregation-prone mutations E46K, H50Q, G51D, A53T, K58N, and G14R, as well as A53E, which is associated with lower aggregation rates (Fig. 5, Table 1). α-Syn mutations, such as A30P, E46K, H50Q, G51D, and A53T, are usually associated with familial forms of PD [79–86]. Specific mutations, such as A53E and G51D [87–89], as well as SNP variants rs11931074, rs3857059, rs3822086, and rs377544 [87, 88], have been associated with MSA. In the case of DLB, no clearly defined genetic determinants were described, likely due to diagnostic challenges, under-recognition of the disease, and a scarcity of well-characterized patient cohorts. Only a few rare variants, such as E46K and rs7681440, have been implicated in DLB. However, their rarity precludes them from serving as reliable diagnostic markers [80, 89].

**Table 1:** Structures of α-Syn filaments carrying familiar mutations

α-Syn	Aggr	Seed	Reported effect	PDB ID	Protofilament interface	Reference
K58N (1–140)	↑	NT	- Faster rate of fibril formation - Subcellular localization in yeast - Increased helical propensity	9HXA	_45_KEGVVHGVATVAE_57_	Al-Azzani et al. (2025)
A53E (1–140)	**↓**	**↑**	- Increased cytotoxicity - Decreased stability of filaments	7UAK	_59_TK_60_	Sun et al. (2023)
A53T (1–140)	**↑**	**↑**	- Increased cytotoxicity - Decreased stability of filaments	6LRQ	_59_TK_60_	Sun et al. (2020)
G51D (1–140)	**↑**	**↑**	- Increased cytotoxicity - Decreased stability of filaments	7E0F	Homotypical steric-zipper _74_VTAVAQ_79_	Sun et al. (2021)
H50Q (36–99)	**↑**	**↑**	- Rapid fibril forming kinetics - Increased cytotoxicity	6PEO–1PF 6PES–2PF	_58_KTKE_61_	Boyer et al. (2019)
E46K (1–140)	**↑**	**↑**	- Increased cytotoxicity - Decreased stability of filaments	6UFR 6L4S 7C1D	_45_KEGVVHGVATVAE_57_ _74_VTAVAQ_79_ _74_VTAVAQ_79_	Boyer et al. (2020) Zhao et al. (2020) Long et al. (2021)
G14R (1–140)	**↓**	NT	- Increased propensity for B-structures	9HGR	_45_KEGVVHGVATVAEKT_59_	Brücke et al. (2025)

*NT *not tested

Familial α-Syn mutations can be classified into four groups according to the protofilament interface regions they form in fibrils prepared from recombinant proteins: (1) mutations promoting the canonical 45–57 interface, similar to WT α-Syn (K58N and E46K); (2). mutations with interface involving residues 58–59 or 58–61 (G14R, H50Q); (3) mutations inducing interface shift toward residues 74–79 (E46K, G51D); and (4) mutations forming very short interfaces around T59–K60 (A53T, A53E).

Mutations K58N (PDB: 9HXA; Fig. 5A) and one of the E46K polymorphs (PDB: 6UFR; Fig. 5B) promote the formation of protofilament interface spanning residues 45–57, a region also involved in wild-type WT α-Syn fibrils. In vitro aggregation studies of the K58N variant revealed the formation of double-protofilament fibrils with a “fold B” (Fig. 5A) [76]. This mutation likely alters the charge at E57, promoting a trans-interaction with K45 of the opposing protofilament, thereby stabilizing double-protofilament assemblies and prolonging the interface (Al-Azzani et al., 2025).

The most extensively characterized familial mutation, E46K, appears in several structural models (PDB: 6UFR, 6L4S, 7C1D; Fig. 5B-D). While 6UFR polymorph retains the canonical WT-like interface involving residues 45–57 stabilized by K45–E57 interaction, the structures 6L4S and 7C1D exhibit a shift in the interface toward residues V74–Q79, forming a novel interface cavity [77, 90, 91]. The E46K substitution alters local electrostatics and hydrogen-bonding networks, especially around residues 45–57, thereby affecting how the protofilaments align and interact. This flexibility enables the formation of multiple energetically favorable packing arrangements, each stabilizing a different polymorph. E46K increases the aggregation rate, seeding, and cytotoxicity of α-Syn. In summary, the structures retaining the 45–57 interface correlate with more efficient aggregation.

In contrast, the G14R double-protofilament fibrils (PDB: 9HGR; Fig. 5E), which extend the interface to residues 58–59, exhibit reduced aggregation rates—possibly due to the altered structural packing at the extended interface. In contrast to K58N, G14R leads to the formation of both double-protofilament and single-protofilament filaments. The interface of double-protofilament fibrils spans 45–59 residues. In the case of a single-protofilament, part of the N-terminus of α-Syn binds the K45-E57 interface, blocks the formation of a double-protofilament, and induces the formation of a single-protofilament [92].

The α-Syn H50Q mutation is characterized by a slightly extended protofilament interface that involves residues 58 and 61. Two polymorph structures were published. The double-protofilament H50Q fibril polymorph (PDB: 6PES; Fig. 5F) is approximately five-fold less abundant in comparison to the more prevalent single-protofilament narrow fibril form (PDB: 6PEO; Fig. 5G) [93]. Similar to the A53T mutation, the H50Q mutation accelerates fibril formation, enhances cytotoxicity, and facilitates the cross-seeding of wild-type α-Syn. Structurally, this mutation disrupts the salt bridge between H50 and E57 that is typically formed in WT fibrils. Instead, it promotes the formation of an intramolecular hydrogen bond between Q50 and K45, a feature observed in both the dominant single-protofilament (6PEO) and the less populated two-protofilament (6PES) forms. In the latter, the interface is defined by a shorter 58-KTKE-61 motif [93].

Interestingly, in comparison to all the abovementioned mutations, G51D fibrils (PDB: 7EOF; Fig. 5H) contain double-protofilaments with right-handed twist. This is unusual among α-Syn fibrils, which are typically left-handed. Similarly to α-Syn E46K filaments, G51D fibrils are stabilized by a non-typical interface 74–79, shifted towards the N-terminus of α-Syn, forming a dry steric-zipper. G51D mutation disrupts the protofilament interface seen in WT α-Syn [90, 91, 94].

A53T and A53E α-synuclein fibrils exhibit a shift in the inter-protofilament interface from the canonical 50–57 region in WT fibrils to a shorter T59–K60 interface, as shown in structures PDB: 6LRQ (Fig. 5I) and 7UAK (Fig. 5J), respectively. This shortened interface may facilitate protofilament dissociation and accelerate fibril propagation, consistent with the early-onset and severe pathology associated with these mutations [95, 96]. While both variants adopt similar fibrillar architectures, A53E filaments form a unique intramolecular salt bridge between K96 and D98 at the C-terminal core. The combination of these structural features likely prolongs the residence time of A53T and A53E α-Syn in toxic oligomeric states, contributing to their elevated cytotoxicity.

Several familial mutations in α-Syn promote distinct fibril architectures with extended protofilament interfaces that enhance aggregation, seeding, and cytotoxicity. Importantly, seeding experiments have shown that mutant α-Syn fibrils can transmit their structural conformation to WT α-Syn, a property critical for RT-QuIC-based or other SAA detection methods of pathogenic strains. These structural insights underscore the assay’s potential sensitivity to fibril strain heterogeneity and support its use in detecting both common and mutation-specific α-Syn conformers.

In summary, structural studies of α-synuclein fibrils isolated post-mortem from human brains demonstrate that mature fibril conformations are highly disease-specific (Fig. 4). By contrast, in vitro–assembled recombinant α-Syn fibrils display substantial structural heterogeneity, as recombinant aggregation readily yields multiple polymorphs that do not fully reproduce the conformations observed in PD, DLB, or MSA (Suppl. Figure 1). For α-synuclein carrying familial PD mutations, structural insights currently rely almost exclusively on fibrils generated from recombinant mutants (Fig. 5). Most of these point mutations alter electrostatic contacts or hydrogen-bonding networks at the protofilament interface, thereby stabilizing specific fibril conformations and enhancing their propagation efficiency. A mechanistic understanding of how familial mutations, as well as post-translational modifications, reshape α-Syn structure is essential for interpreting how these molecular alterations influence seeding behavior in RT-QuIC assays.

### The Role of Post-translational Modifications in α-Synuclein Filament Pathology

**Fig. 6:**
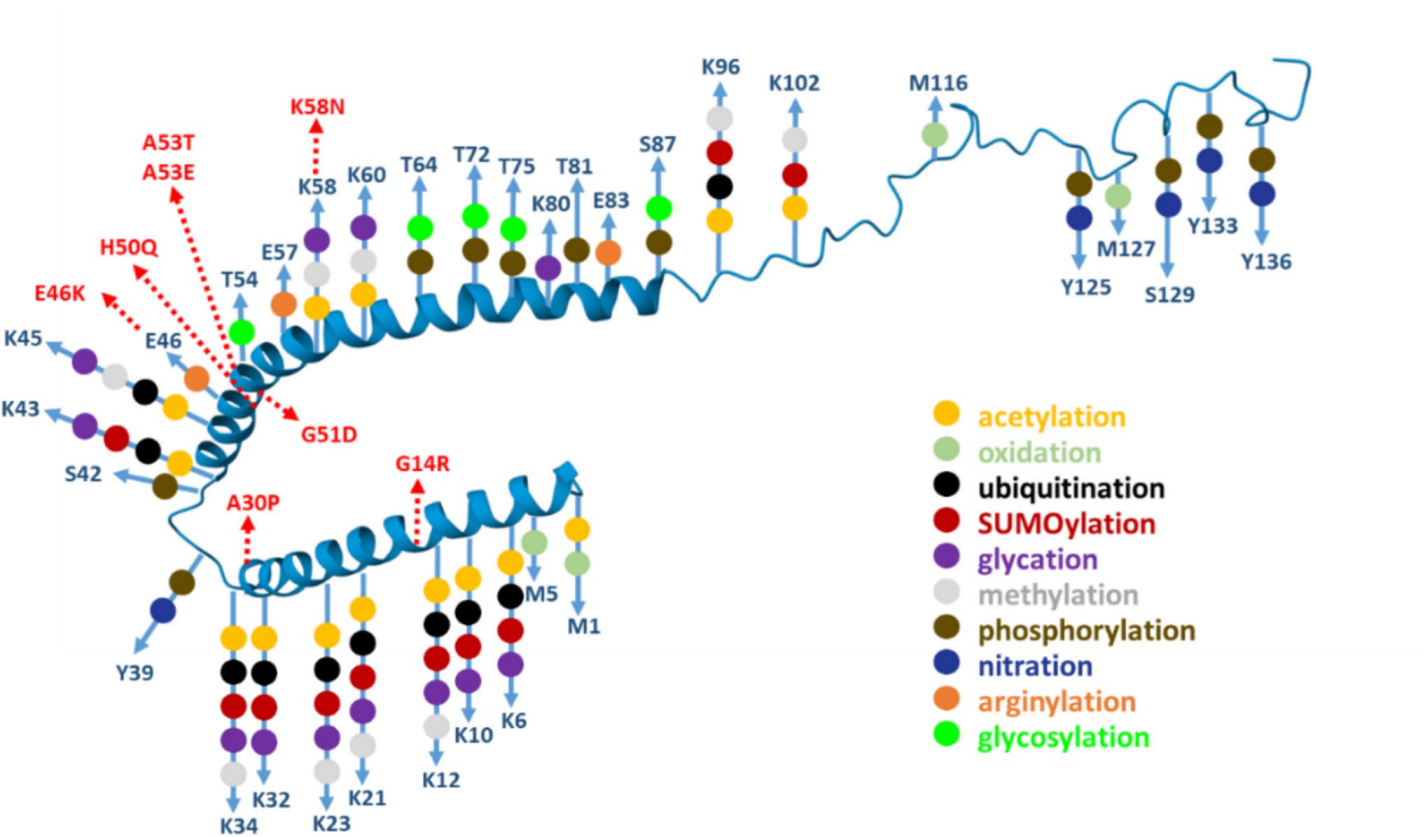
α-Syn post-translational modifications. α-Syn undergoes numerous post-translational modifications (PTMs). In the scheme, sites of PTMs and also familiar mutations are highlighted on the structure of micelle-bound α-Syn (PDB: 1XQ8)

α-Syn undergoes multiple post-translational modifications (PTMs), such as acetylation, phosphorylation, glycosylation, ubiquitination, SUMOylation, nitration, oxidation, and truncations (Fig. 6. These modifications often act in concert to modulate α-Syn folding, aggregation, and degradation pathways, thereby shaping disease progression. Detection of PTMs of α-Syn thus represents an attractive approach in identifying a potent and specific biological marker for synucleinopathies [97]. Among the most studied PTMs, phosphorylation and truncation exert remarkably opposing yet complementary effects. Phosphorylation at Ser129, abundant in Lewy bodies, promotes a more soluble yet aggregation-competent intermediate that enhances oligomer toxicity while interfering with proteasomal degradation. In contrast, C-terminal truncation removes acidic residues that typically stabilize the native conformation, accelerate fibril nucleation, and facilitate the formation of compact β-sheet–rich assemblies. Ubiquitination often targets these truncated or phosphorylated species for clearance, whereas nitration and acetylation can hinder degradation and stabilize toxic oligomers by blocking lysine residues essential for ubiquitin conjugation. The majority of α-Syn isolated from both healthy and diseased brain tissue is N-terminally acetylated [44, 98]. N-terminal acetylation modulates the protein’s conformation, membrane affinity, and aggregation behavior and is considered essential for reproducing physiologically relevant fibril structures in vitro [41, 44]. Cross-talk between PTMs, therefore, defines whether α-Syn remains in a reversible state or shifts irreversibly toward pathogenic aggregates.

A significant hallmark in Lewy bodies and glial cytoplasmic inclusions is phosphorylation of α-Syn at serine 129 (pS129). In a healthy brain, only approximately 4% of α-Syn is phosphorylated at Ser129, whereas in synucleinopathies, this percentage rises to > 90% [98, 99]. However, recent data suggest that pS129 modification occurs secondarily to α-Syn accumulation and may actually inhibit α-Syn cytotoxicity and aggregation, thereby potentially serving as a protective mechanism [100]. Despite its prevalence, the structure of the pS129 α-Syn remains unknown.

Structure of fibrils with phosphorylation on another residue, Y39 (pY39; PDB: 6L1T, 6L1U; Suppl. Figure 2A, B; Table 2) was solved. pY39 leads to increased cytotoxicity of fibrils, increased propagation rate in cortical neurons, and increased resistance to proteolytic enzymes. In comparison to non-phosphorylated α-Syn, recombinant pY39 α-Syn filaments are stabilized by the interaction of the N-terminal part directly with the C-terminal part, increasing the stability and robustness of pY39 fibrils and thus higher resistance to proteolysis [101].

**Table 2:** Comparison of α-Syn fibrils with PTMs

α-Syn	Aggr	Seed	Reported effect	PDB ID	Protofilament interface	Reference
pY39 1–140	**↑**	**↑**	- Increased cytotoxicity - Increased fibril propagation in neurons	6L1T – 2PF 6L1U – 3PF	_57_EK_58_ _57_EK_58_ – E_46_ (PF I – PF II – PF III)	Zhao et al. (2020)
gS87 1–140	**↓**	**↓**	- Decreased cytotoxicity - Decreased fibril propagation in neurons	8JEX		Hu et al. (2024)
pS87 1–140	**↓**	**↓**	- Decreased cytotoxicity - Decreased stability of fibrils - Decreased fibril propagation in neurons	8JEY	_1_MDVFMKG_7_—_36_GVLYV_40_	Hu et al. (2024)
gS87 1–140	**↓**	**↓**	- Decreased fibril propagation in neurons - Inhibition of aggregation initiation by maintaining soluble α-Syn	8GF7	_57_EK_58_	Balana et al. (2021)
41–140 N-trunc	**↓**	**↓**	- Unable to seed WTα-Syn - Reduction of membrane binding	7LC9	_60_ K-F_94_ (PF I)—_62_Q-I_88_ (PF II)	McGlinchey et al. (2021)
∆2–7 (1, 8–140)	**↓**	**↓**	- Decreased cytotoxicity	8QPZ	_50_HGVATVAEK_58_	Dewison et al. (2024)
1–122 C-trunc	**↑**	**↑**	- Tighter packaging of amyloid-core	6OSL	_50_HGVATVAE_57_	Ni et al. (2019)
1–103 C-trunc	**↑**	**↑**	- Tightest packaging amyloid-core - Increased stability of filaments	6OSM	_50_HGVATVAE_57_	Ni et al. (2019)
1–121 C-trunc	**↑**	**↑**	- Increased cytotoxicity	6H6B	_50_HGVATVAE_57_	Guerrero-Ferreira et al. (2018)

On the other hand, both phosphorylation (PDB: 8JEY; Suppl. Figure 2C) and glycosylation (PDB: 8JEX; Suppl. Figure 2D) of S87 (pS87 and gS87) decreased neurotoxicity and seeding capability of the fibrils. These fibrils have a different fold in comparison to WT fibrils without these PTMs and are different from each other. In structure 8JEX, an intramolecular interaction between gS87 and K80, T81, V82, I88, and A89 residues and an additional intramolecular E61-K80 salt bridge form, which stabilizes the protofilaments, was identified. The protofilaments are not creating a stable interface through intermolecular interactions. Instead, fibrils formed by pS87 directly involve the N-terminal region in the fibril core, and the filaments are stabilized by intramolecular salt bridges of K6-E20 and D2-K21. However, the exact relation of these structural changes and their decreased effect on pathology has not been described [102]. gS87 might be able to promote interactions of α-Syn with chaperons and other molecules, destabilizing fibril structures and thus maintaining α-Syn in soluble form [103]. Interestingly, another gS87 α-Syn filament structure is also available (PDB: 8GF7; Suppl. Figure 2E) that is entirely different from 8JEX. In 8GF7, double-protofilaments are stabilized by E57-K58 electrostatic interactions, and gS87 does not interact with any neighboring residues.

Other PTMs, ubiquitination, and SUMOylation affect α-Syn degradation pathways. Ubiquitination is a PTM that regulates α-Syn degradation through the ubiquitin–proteasome system and the autophagy-lysosomal pathway. In synucleinopathies, ubiquitinated α-Syn is abundantly found in Lewy bodies and glial cytoplasmic inclusions, suggesting a failure of protein clearance mechanisms. However, in general, ubiquitination has a positive effect, as it inhibits aggregation and toxicity and promotes the clearance of α-Syn [97]. Mono- and di-ubiquitination has been detected at almost every α-Syn lysine residue (K6, K10, K12, K21, K23, K32, K34, K43, K45, K58, K60, K96, and K102). But residues 45, 58, and 60 are the most abundant ubiquitination sites and are important in α-Syn degradation [104]. In contrast, glycation and SUMOylation may interfere with ubiquitination, leading to decreased α-Syn degradation and accumulation in neurons. While ubiquitination and SUMOylation are known to modulate α-Syn aggregation and turnover, their precise impact on the molecular structure of α-Syn fibrils remains to be elucidated.

Also, nitration and oxidation increase the toxicity and aggregation of α-Syn. Nitration is a non-enzymatic PTM that modifies tyrosine residues Y39, Y125, Y133, and Y136 in α-Syn [105, 106]. Nitrated α-Syn oligomers are more toxic than non-modified forms, triggering microglial activation, neuroinflammation, and oxidative stress, leading to dopaminergic neuron loss in PD models [107]. Nitration of α-Syn promotes its dimerization and oligomerization, and nitrated α-Syn is enriched in Lewy bodies and glial cytoplasmic inclusions, particularly in DLB and MSA brains. Y39 nitration negatively impairs membrane binding of α-Syn, reducing its physiological function in synaptic vesicle trafficking [107]. Oxidation of α-Syn occurs due to reactive oxygen species (ROS), modifying methionine residues M1, M5, M116, and M127 and affecting protein conformation and aggregation. Methionine oxidation prevents fibril formation, stabilizing oligomeric α-Syn species, which may be more toxic than fibrillar forms [108]. Oxidized α-Syn interacts with cytochrome c, promoting mitochondrial dysfunction and apoptosis in dopaminergic neurons [109]. Like ubiquitination and SUMOylation, nitration and oxidation have not yet been fully resolved in high-resolution fibril structures (e.g., via cryo-EM). Most structural data come from biochemical, biophysical, and lower-resolution techniques (e.g., NMR, CD, MS), rather than atomic-resolution models of fibrils carrying these modifications.

Truncations and cleavage are among the most frequent α-Syn PTMs and usually enhance its fibrillation. Up to 30–50% of α-Syn in Lewy bodies is found to be C-terminally truncated, particularly in forms lacking residues beyond residue 115. C-terminal truncations promote β-sheet-rich oligomer formation, accelerate Lewy body pathology, and impair α-Syn clearance by proteolytic systems [110]. Truncated α-Syn species have been observed in post-mortem brain tissue from patients with PD, DLB, and MSA [111–114]. Different proteases have been implicated in cleaving α-Syn, including calpain-1 [118], neurosin [119], matrix metalloproteases (MMPs) [120], cathepsin D [121], the 20S proteasome [115, 116], and caspase-1 [117].

Several structures of C-terminally truncated α-Syn are already available. Truncated filament 1–122 (PDB: 6OSL; Suppl. Figure 2F) and 1–103 (PDB: 6OSM, Suppl. Figure 2G) preserve the core protofilament interface (H50–E57) but induce conformational adaptations in surrounding regions, involving K58, E61, and V74–V82 [118]. This region is further stabilized by intermolecular interactions between E57 on one protofilament and H50 and K45 on the other protofilament. The truncation 1–103 exhibits additional stabilizing interactions, including an E61–T72 hydrogen bond and an E46–K80 salt bridge, which likely bring the N-terminal region into proximity to the fibril core. This correlates with the increased protease resistance of these filaments. Interestingly, C-terminal truncations also promote fibril twisting, with the degree of twist rising in proportion to the extent of truncation [118]. Filaments 1–121 are characterized by the formation of class 1 A (PDB: 6H6B; Suppl. Figure 2H) polymorphs [74]. Fibrils are composed of two identical protofilaments, and the interface region spans residues 50–57 and involves hydrophobic interactions. All characterized C-terminally truncated fibrils efficiently cross-seed full-length α-Syn, inducing a more twisted polymorph, demonstrating the ability of truncated forms to template distinct conformations.

While C-terminal truncations are described to promote aggregation, N-terminal truncations are, on the other hand, associated with decreased rate of propagation and seeding activity, suggesting that the N-terminal region can be crucial in the assembly of α-Syn filaments. N-truncated α-Syn fragments 14–140, 36–140, and 41–140 have shown decreased ThT signal when compared to WT full-length α-Syn. Cross-seeding further revealed that only 14–140 α-Syn can promote aggregation of WT α-Syn. In comparison, cross-seeding by WT α-Syn was successful for both 14–140 and 36–140 α-Syn samples and not for 41–140 α-Syn. The structure of 41–140 α-Syn (PDB: 7LC9; Suppl. Figure 2I) was subsequently solved by cryo-EM with one protofilament comprising E46-K96 residues and the second protofilament E61-D98 residues. The protofilaments are interacting via the K60-F94 – Q62-I88 interface by an intermolecular K80-E83 salt bridge and a Q62-Q62 hydrogen bond [119]. It has been speculated that the decreased density of the N-terminal region is due to its higher flexibility, similar to residues 99–140 of the C-terminal region. Indeed, deletion of residues 2–7 (∆2–7; PDB: 8QPZ; Suppl. Figure 2J) also leads to decreased aggregation rate, decreased ability of seeding WT α-Syn, and reduced cytotoxicity in *C. elegans*, confirming the involvement of the N-terminal region in the maturation of α-Syn fibrils [120].

Also, other factors, such as gene multiplication, environmental exposures, certain infections, and age-related decline in cellular homeostasis, contribute to α-Syn aggregation. Most likely, the aggregation of α-Syn in synucleinopathies results from a multifactorial interplay among genetic factors, environmental triggers, PTMs, and age-related declines in cellular maintenance mechanisms. Nevertheless, PTMs of α-Syn offer promising avenues for early detection and therapeutic intervention in PD, DLB, and MSA and may serve as potent biomarkers. Currently, pS129 α-Syn is being introduced to the market as a biochemical marker for synucleinopathies. Quantitative immunoassays and other ultrasensitive platforms (e.g., single-molecule array, SIMOA, and Meso Scale Discovery Electrochemiluminescence, MSD ECL) allow the detection of pS129 α-Syn in cerebrospinal fluid, plasma, and skin biopsies. Diagnostic panels incorporating phosphorylated α-Syn for PD testing are already commercially available. However, these assays are not yet routinely used in diagnostics. So far, only one was CE-marked in Europe and is under FDA review (Syn-One Test). A combined assay integrating phosphorylated α-Syn detection with RT-QuIC, or other seed amplification assays, could enhance the robustness of diagnostics [121–123].

### From Structure to Signal: Translating α-Synuclein Biochemistry into Diagnostic Amplification Assays

The advances in elucidating the structural polymorphism and biochemical behavior of α-Syn provide the essential basis for RT-QuIC. The same molecular principles that are involved in monomer misfolding, nucleation, and fibril elongation drive the kinetics of RT-QuIC. Structural studies have shown that variations in protofilament interfaces, β-sheet stacking, and post-translational modifications directly affect the seeding capacity, fibril morphology, and propagation behavior of α-Syn. These molecular characteristics define how efficiently a pathological seed can convert recombinant substratein vitro, determining the lag phase, growth rate, and ThT fluorescence profile observed in RT-QuIC readouts. Truncated α-Syn, when used as a substrate, may, for instance, enhance the sensitivity of RT-QuIC or SAA detection methods.

In addition, optimizing assay conditions, such as substrate sequence, ionic composition, and temperature, can selectively favor the amplification of certain α-Syn strains, effectively recapitulating structural features observed in patient-derived filaments. RT-QuIC reflects the structural biochemistry of α-Syn, and nanoscale conformational diversity is transformed into measurable kinetic signatures. In the following chapters, we will discuss these aspects of RT-QuIC.

## MAD—Detected: Fundamentals and Recent Advances of RT-QuIC

Currently, diagnosis relies on clinical assessment and neuroimaging, which often lack specificity, especially in early disease stages [124]. Definite diagnosis typically requires post-mortem confirmation of α-Syn aggregates, such as Lewy bodies or glial cytoplasmic inclusions. Misdiagnosis is common due to symptom overlap among disorders like PD, DLB, and MSA. Efforts are now focused on identifying reliable biomarkers in cerebrospinal fluid (CSF), blood, and tissues, including pathological forms of α-Syn [122]. The methods for identifying α-Syn oligomers or aggregates are summarized in Table 3 below, but none are used routinely in diagnostics. Although promising, current biomarker studies remain inconsistent. There is an urgent need for standardized protocols and involvement of larger cohorts.

**Table 3:** Comparison of sensitive detection methods

Assay	Seeding mechanism/detection principle	Sensitivity	Specificity	Key applications
RT-QuIC	Shaking-induced fibril conversion with ThT fluorescence	High	Very high	Detection
Nanobody-based ultrasensitive immunoassay (NULISA)	Nanobody-based immunodetection of α-Syn aggregates	Very high	Very high	Detection
Single-molecule array (SIMOA)	Digital immunoassay with single-molecule resolution	High	Very high	Detection
ELISA for α-Syn oligomers	Antibody-based detection	Moderate	High	Screening tool
Protein misfolding cyclic amplification (PMCA)	Sonication-induced fibril fragmentation	Very high	Moderate	Detection
Mass spectrometry-based methods	Direct protein analysis	High	High	Biomarker discovery

Very high (> 95%), high (> 90%), and moderate (> 80%)

The Real-Time Quaking-Induced Conversion (RT-QuIC) assay has emerged as a valuable method to detect misfolded α-Syn aggregates. RT-QuIC is a highly sensitive assay for detecting aggregated proteins, initially developed for prion diseases. It measures the conversion of soluble proteins into an aggregated state, which can be quantified using fluorescence detection [125]. The RT-QuIC method’s utility in identifying misfolded proteins has generated interest in its application beyond prion diseases [126, 127]. Researchers have adapted the RT-QuIC technique to target α-Syn aggregates in biological samples specifically [128–131]. By modifying the assay conditions, including the use of various buffers and substrates, RT-QuIC can effectively amplify and detect even low concentrations of pathological α-Syn in CSF and other relevant fluids or tissues. RT-QuIC is part of a broader family of SAAs used in neurodegenerative disease research. A comparison with related techniques highlights its advantages for clinical use (Table 3). The key advance of RT-QuIC lies in its ability to provide rapid results compared to other methods and its potential to use non-invasive samples.

**Fig. 7:**
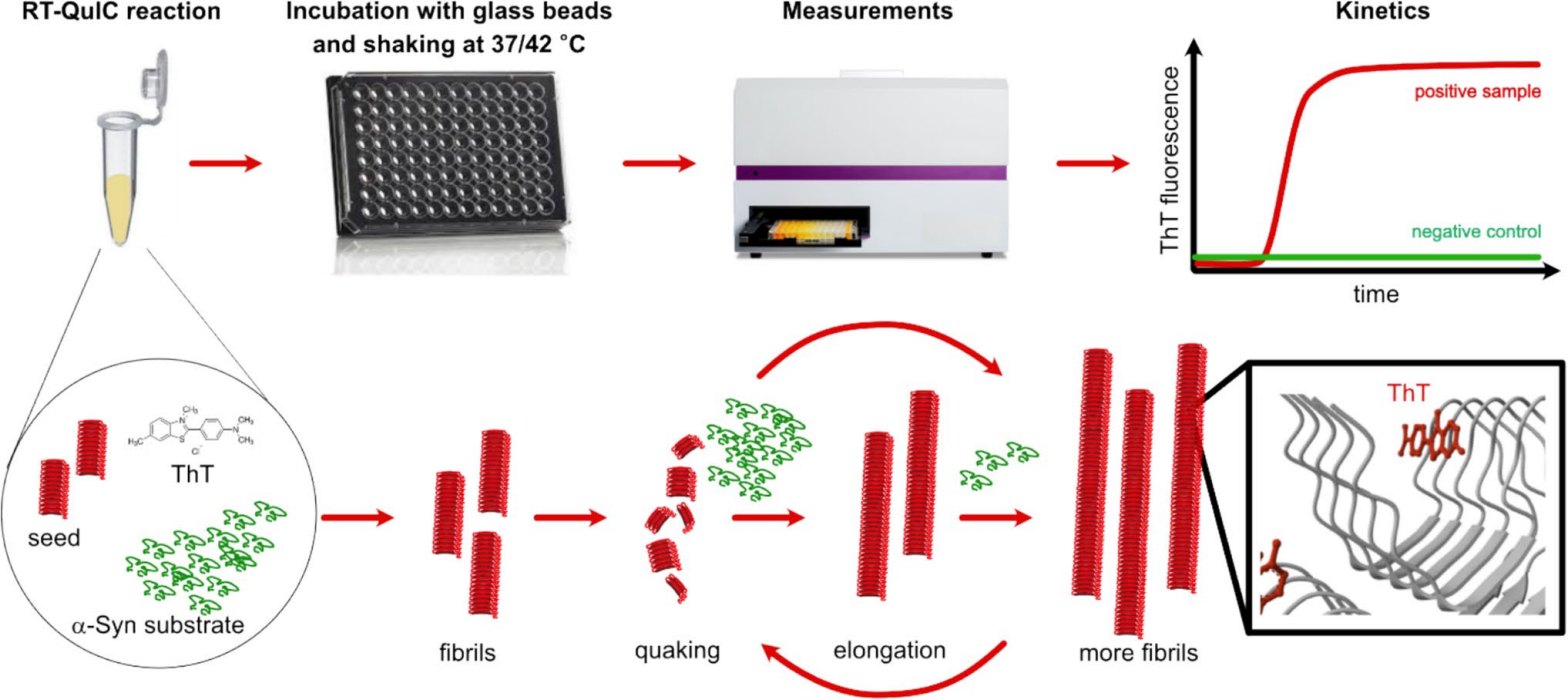
Key steps in RT-QuIC assay. Recombinant human α-Syn monomers serve as a substrate in a reaction containing buffer, salts, and ThT. The sample (CSF, skin biopsy homogenate, or another biospecimen) is added to the reaction mixture as a potential source of misfolded α-Syn seeds. The reaction mixture is then subjected to alternating cycles of shaking and resting at a controlled temperature, typically at or above 37 °C. Shaking facilitates fibril fragmentation, generates new seeding surfaces, and promotes signal amplification. ThT fluorescence is measured at regular intervals to track amyloid fibril formation. Positive samples exhibit a steep increase in fluorescence, indicating the presence of pathogenic α-Syn species. The faster the fluorescence increases, the higher the concentration of seeding-competent α-Syn aggregates in the sample. Negative samples show delayed or absent fluorescence signals, consistent with the lack of misfolded α-Syn. Adapted from Peña-Bautista et al. (2023). α-Syn fibril structure with bound ThT is from the PDB depository 8X7B

RT-QuIC is based on the concept that misfolded α-Syn can act as a seed to induce conformational changes in normal monomeric α-Syn (substrate), leading to fibril formation in a controlled in vitro environment. The amplification process follows a sigmoidal kinetic curve, composed of three key phases (Fig. 7). A lag phase during which the native recombinant α-Syn remains in a monomeric state with minimal aggregation, followed by an exponential phase in which pathological α-Syn seeds accelerate fibrillation, forming structured aggregates. And finally, a plateau phase as the reaction reaches saturation after all available α-Syn monomers convert into fibrils [132]. During this process, ThT, a fluorescent dye that binds to β-sheet-rich amyloid structures, is used to monitor fibril formation in real-time [133].

While α-Syn protein holds promise as a biomarker for PD diagnosis and prognosis, further research is needed to establish a consistent, reliable approach. According to the recent meta-analysis studies, the diagnostic accuracy of the RT-QuIC assay is high, with a pooled specificity of 88–95% and a sensitivity versus controls of 95% [132, 134, 135]. Usually, cerebrospinal fluid (CSF) is included as a sample. There is also higher variability in the accuracy of the assay for different synucleinopathies, e.g., for PD 85–98% sensitivity and 90–98% specificity, for DLB 90–98% sensitivity and 92–99% specificity, and for MSA 50–70% sensitivity and 90–95% specificity were reported (Table 4).

**Table 4:** Comparison of meta-analysis studies on RT-QuIC

Aspect	Grossauer et al. (2023)	Peña-Bautista et al. (2023)	Wang et al. (2022)
Scope	All synucleinopathies (PD, DLB, MSA, prodromal stages)	DLB-focused (CSF and peripheral tissues; co-pathology stratified)	Lewy body diseases broadly (DLB, PD, PDD, PAF)
Studies included	22	25	16
Sample types	CSF only	CSF, skin, brain, olfactory mucosa, glands	CSF only
Pooled sensitivity	0.88 (synucleinopathies vs. controls)	0.94 (DLB vs. controls)	0.91 (Lewy body diseases vs. controls)
	0.91 (synucleinopathies with LB pathology vs. controls)		
		0.95 (DLB vs. AD)	
Pooled specificity	0.95 (synucleinopathies vs. controls)	0.96 (DLB vs. controls)	0.95 (Lewy body diseases vs. controls)
	0.96 (synucleinopathies with LB pathology vs. controls)	0.88 (DLB vs. AD)	
Prodromal stages	Sensitivity 0.74	Sensitivity 0.78	Sensitivity 0.76
	Specificity 0.93	Specificity: 0.95	Specificity N/A
MSA analysis	Sensitivity low (30%) for RT-QuIC; PMCA more sensitive	N/A	N/A
Technical parameters	No technical subgrouping	Detailed analysis: temp, buffer, pH, salt, SDS, shaking, etc	Detailed analysis: bead size, shaking, temp, buffer conc., etc
Key technical insights	Protocol heterogeneity recognized	Buffer pH, NaCl, SDS, shaking did not significantly affect results	Optimal setup 0.8/1.0 mm beads, 42 °C, 400 rpm, 40 mM phosphate buffer
Co-pathology and genetics	N/A	Assessed AD co-pathology and GBA/APOE genotypes	N/A
AUC (diagnostic accuracy)	N/A	N/A	AUC = 0.97
Assay comparison	RT-QuIC and PMCA	Only RT-QuIC	Only RT-QuIC
Outcome	High utility in synucleinopathies, especially DLB; limitations in MSA	Strong diagnostic value for DLB incl. prodromal; co-pathology influence manageable	Accurate, rapid, and clinically applicable for Lewy body diseases

A critical challenge in RT-QuIC-based diagnostics is its variable sensitivity for MSA, which is often lower compared to PD and DLB. This reduced diagnostic accuracy reflects structural and biochemical differences between the underlying α-Syn strains. In MSA, α-Syn aggregates usually form within glial cytoplasmic inclusions rather than neuronal Lewy bodies, producing fibrils with distinct protofilament interfaces, higher stability, and altered seeding kinetics [136, 137]. These strain-specific features result in weaker or delayed RT-QuIC fluorescence responses compared to those observed with PD or DLB seeds. Recent optimization studies have begun to address these limitations by using of A53T or truncated α-Syn substrates, fine-tuning the ionic strength, and cofactors such as heparin or poly-anionic enhancers [138–140]. Continued optimization of assay parameters and substrate engineering has the potential to improve both MSA sensitivity and the overall clinical applicability of RT-QuIC.

Notably, RT-QuIC has shown higher sensitivity and specificity in distinguishing synucleinopathies from other neurodegenerative disorders, like Alzheimer’s disease (AD), than in distinguishing synucleinopathies from each other. As reported in the meta-analysis by Peña-Bautista et al. (2023), RT-QuIC demonstrates high accuracy in differentiating AD from DLB, with 95% sensitivity and 88% specificity. However, specificity was very low for PD vs. DLB (11%), indicating poor differentiation between the two of them using RT-QuIC [132].

RT-QuIC also holds significant diagnostic value in prodromal synucleinopathies, where molecular pathology precedes clinical manifestation by many years. Several studies show that α-Syn seeding activity can be detected in individuals with idiopathic REM sleep behavior disorder (iRBD). iRBD is the most potent clinical precursor of PD and DLB, and it starts well before the onset of disease or cognitive decline. In a longitudinal cohort, CSF RT-QuIC was positive in the majority of iRBD patients and predicted conversion to synucleinopathy with high accuracy, often outperforming dopamine transporter imaging [141]. More recently, highly sensitive assays applied to the DeNoPa (“De Novo* Parkinson*”) cohort confirmed α-Syn seeding activity in 93% of iRBD cases [142]. Promising results were obtained with peripheral tissue RT-QuIC assays, particularly olfactory mucosa and skin. They achieved 45% sensitivity with mucosa and 77% with skin samples, and 90% and 98% specificity, respectively, in patients with iRBD [143, 144]. Diagnostic utility extends also to pure autonomic failure (PAF), another prodromal syndrome on the DLB spectrum. RT-QuIC reliably identified α-Syn seeds in CSF [145]. Collectively, these findings establish RT-QuIC as a powerful molecular tool for uncovering prodromal synucleinopathy, enabling early detection across clinical phenotypes.

## Technical Optimizations and Standardization Challenges

RT-QuIC has great promise for diagnosing synucleinopathies by detecting misfolded α-Syn, even in early stages. However, there are still some technical challenges to overcome. Multiple factors, including reaction conditions, substrate, inter-laboratory variability, and lack of automation, influence the assay’s sensitivity, specificity, reproducibility, and scalability. Biological variability, such as seed concentration and its heterogeneous distribution across tissues, sample dilution effects, the presence of inhibitory components in samples, and the quality of the substrate, can result in false-negative results even when pathogenic α-Syn is present. Furthermore, the kinetics and efficiency of RT-QuIC depend on sample type, reaction conditions, including temperature, shaking conditions, buffer composition, and pH. The precise mechanisms underlying the effects of the reaction parameters remain unclear and are likely multifactorial. Higher temperatures probably enhance entropy and the frequency of collisions between α-Syn seeds and substrate, thereby accelerating the nucleation process [146]. Shaking likely facilitates both homogeneous mixing of the reaction components and mechanical fragmentation of fibrils, producing additional seeding-competent ends [147]. The presence of Triton X-100 or other detergents may help disperse seeds more uniformly or alter monomer conformation, making them more amenable to conversion. Probably the most critical variable in the assay represents the substrate type and quality. Collectively, these factors highlight the importance of ongoing optimization efforts to expand the dynamic range and strain sensitivity of the assay.

### Sample

Expanding RT-QuIC beyond CSF to non-invasive samples represents a major advancement toward making α-Syn detection more accessible for clinical diagnostics. Recent studies have validated RT-QuIC for α-Syn detection in peripheral tissues, including the olfactory mucosa, which can be obtained via nasal swabs, making it a promising and minimally invasive biomarker source. On these samples, RT-QuIC has about 90% sensitivity and 95% specificity for PD and DLB [132]. Another promising specimen is the skin, which can provide misfolded α-Syn present in cutaneous nerve fibers. Sensitivity is 70–100% for PD and 60–80% for MSA [148, 149]. The ultimate, very easily accessible source of samples for detection is blood- and plasma-derived exosomes. So far, the RT-QuIC used on exosomes performs with lower sensitivity (50–75%) due to low α-Syn concentrations. However, improvements in exosomal enrichment methods are increasing their diagnostic utility [150, 151]. Also, urine has recently been explored as a non-invasive biofluid for RT-QuIC, offering a readily accessible sample type. Detection of pathological α-Syn in urine showed very high specificity (100%) but limited sensitivity (22%) and reproducibility [152]. While CSF remains the gold standard for reliably detecting pathologic α-Syn, other samples, such as skin, olfactory mucosa, blood, and urine, are showing promise. However, routine usage of less invasive samples still faces technical limitations and requires further optimization.

### Temperature

RT-QuIC assays for α-Syn detection typically operate at 37 to 45 °C to optimize fibril formation while maintaining protein stability. Temperatures above 45 °C are not commonly used in the α-Syn RT-QuIC but are found in prion RT-QuIC protocols. Higher temperatures have been shown to enhance prion protein amplification rate and shorten the lag phase [153, 154]. However, this can also increase background noise, increase nonspecific ThT fluorescence, and ultimately increase the risk of false-positive results. Conversely, lower temperatures (e.g., 37–40 °C) promote slower, more controlled aggregation, improving assay specificity but potentially compromising sensitivity by extending lag phases and diminishing the overall signal amplitude. Several studies suggest that an intermediate temperature of 42 °C represents a practical compromise, balancing seeding kinetics, assay duration, and specificity [130, 145, 155].

### Shaking

Intermittent shaking—typically between 400 and 800 rpm—interspersed with rest periods was suggested to be a critical mechanical attribute of RT-QuIC assays. This shaking regime facilitates the fragmentation of growing α-Syn fibrils. It generates new fibril ends, which act as additional seeds, thereby enhancing amplification efficiency and signal kinetics [132]. The quaking motion accelerates the conversion of monomeric α-Syn into amyloid fibrils by increasing seed-monomer interactions and promoting nucleation-dependent polymerization within the reaction well. However, substantial variability in shaking protocols, e.g., amplitude, orbital diameter, duration, frequency, and rest interval settings, exists across different laboratories and equipment platforms. This inconsistency has been shown to influence key readout parameters, such as lag phase duration, ThT fluorescence amplitude, and aggregation rate, which in turn affect diagnostic reproducibility and inter-laboratory comparability [132]. Most commonly, shaking conditions with 1 min shaking/1 min rest [130, 145, 155–158] or 1 min shaking/14 min rest [129, 141, 159] are being used.

Similar findings were reported in RT-QuIC assays for prion detection, where shaking conditions significantly impacted reaction kinetics [153]. Excessive shaking may cause non-specific aggregation or shear-induced misfolding, while insufficient shaking can delay or reduce the amplification signal. Therefore, it is essential to optimize shaking parameters to apply consistent mechanical energy. This can balance fibril fragmentation with structural integrity while minimizing artifacts and false positives.

### Beads

Among the critical experimental parameters are the properties of the beads used in RT-QuIC. Beads amplify mechanical forces during shaking, facilitate fibril breakage, and increase the number of nucleation sites available for further seeding [130, 132]. The material, bead surface chemistry, and the size can significantly affect the reaction. Studies comparing glass, silica, and zirconium beads have demonstrated significant variability in lag phase duration and total ThT signal intensity [131, 160]. The 0.5–1.0 mm silica or glass beads provided the most consistent amplification for α-Syn seeds in cerebrospinal fluid [132, 134]. Larger or denser beads increase shear forces but can also induce non-specific aggregation or reduce reproducibility, whereas smaller beads promote homogeneous energy distribution but may limit fragmentation efficiency. Optimal results are typically achieved with six 0.8 mm silica beads [132]. However, manual bead loading remains a significant bottleneck in the assay. It is a laborious, low-throughput, and ergonomically challenging step that is still not automated in most laboratories. Inconsistent bead placement contributes to well-to-well variability and limits the scalability of RT-QuIC for clinical use. Development of automated bead-dispensing and plate-handling systems will therefore be crucial for achieving assay standardization and industrial translation.

### Buffer Composition and pH

The buffer composition, especially pH, is a critical parameter in RT-QuIC assays. It directly influences the efficiency and reproducibility of α-Syn fibril formation. Buffers should maintain the ionic strength, pH stability, and protein solubility throughout the amplification process. This is essential for controlled nucleation and elongation of amyloid fibrils. The most commonly used buffers are phosphate-buffered saline (PBS) [132] and HEPES buffer [160], typically adjusted to a slightly alkaline pH range of 7.4–8.4. But also, lower pH and PIPES buffer were used in several studies [161–163]. pH variations might impact fibril kinetics, ThT fluorescence intensity, and lag phase duration.

The presence of additional cofactors, such as heparin, divalent metal ions like Cu^2^⁺ and Fe^3^⁺, or detergents, can further modulate α-Syn aggregation. Heparin is known to accelerate fibril nucleation by stabilizing β-sheet-rich oligomeric intermediates, effectively reducing the lag phase and enhancing seeding [164]. Metal ions such as Cu^2^⁺ and Fe^3^⁺ interact with specific α-Syn residues, promoting cross-linking, aggregation nucleation, and alterations in fibril morphology [165, 166]. Low concentrations of detergents, particularly SDS, are commonly used in α-Syn RT-QuIC assays with CSF samples [129, 130]. Detergents enhance the uniform dispersion of α-Syn monomers and pathological seeds, reduce surface tension, and promote consistent interactions between seeds and monomers. Detergents facilitate more efficient fibril amplification while also preventing spontaneous aggregation, thereby increasing the sensitivity and specificity of the assay.

While additives can enhance RT-QuIC sensitivity, they may also introduce variability by favoring specific α-Syn strains or altering fibril polymorphism, complicating assay reproducibility across laboratories. Standardization of buffer composition, pH adjustment, and co-factor concentration is essential to achieve high diagnostic accuracy, reproducibility, and inter-laboratory comparability in RT-QuIC-based biomarker assays for synucleinopathies.

### Substrate

Use of mutant or truncated α-Syn variants as substrates can also significantly alter assay sensitivity and specificity and has potential for differential diagnosis of PD, MSA, and DLB. Pathological α-Syn from MSA patient samples, but not from PD or DLB samples, was shown to aggregate α-Syn with mutation A53T in vivo [136, 167, 168]. Similarly, α-Syn with the E46K mutation enhances DLB α-Syn aggregation but prevents MSA α-Syn aggregation [137]. Other mutations, H50Q and K23Q, alter fibril formation kinetics. K23Q mutation results in α-Syn fibrillization kinetics comparable to wild-type α-Syn in the presence of a seed but displays slower spontaneous fibril formation in its absence [169]. The H50Q mutation and C-terminally truncated α-Syn, especially beyond residue 115, promote α-Syn aggregation in vitro by decreasing monomer solubility and shortening the fibril formation lag phase [82, 112–114, 138, 170, 171]. Such variants of α-Syn, when used as a substrate in RT-QuIC, might not only shorten assay time but also increase assay sensitivity, especially in prodromal cases.

Recently, also highly sensitive sdRT-QuIC (same-day Real-Time Quaking-Induced Conversion) assay was reported for detecting misfolded α-Syn seeds with significantly shortened assay time [155]. Compared with traditional RT-QuIC assays that require ≥ 48 h, sdRT-QuIC can be completed in less than 12 h for brain, skin, and intestinal mucosa samples. In this assay, the K23Q α-Syn variant was used to reduce spontaneous fibril formation, and a specific combination of Triton X-100 concentration, temperature, and shaking speed was optimized to enhance assay performance [155]. The addition of non-ionic detergents was shown to decrease RT-QuIC assay duration, also in applications with CSF specimens [123, 172].

Interestingly, according to a recent meta-study by Peña-Bautista et al., the variations of temperatures, time of shaking-rest cycles, shaking speeds, NaCl concentration, SDS concentration, buffer concentration, and buffer pH do not have a significant effect on the sensitivity or specificity of the assay [132]. However, the combined fine-tuning of individual parameters could lead to significant improvements, influencing both the overall duration and the reproducibility of the assay [135, 155].

To improve cross-laboratory reproducibility, collaborative studies comparing RT-QuIC performance are essential and may lead to global harmonization of conditions used in clinical and research applications. An important step is the establishment of standardized reference materials (e.g., certified substrate and misfolded α-Syn standards), which will allow for quality control and inter-laboratory calibration. To transition RT-QuIC from research settings into clinical diagnostic laboratories, improvements in assay automation and scalability are needed. This might include 384-well plate platforms, automated RT-QuIC systems (both sample-handling and plate-reading systems), or the development of microfluidic-based RT-QuIC.

Beyond synucleinopathies, α-Syn is now used as a relevant biomarker in broader neurodegenerative contexts. In 2023, the National Institute on Aging– Alzheimer's Association (NIA–AA) research framework for AD included α-Syn species in CSF as a potential marker of co-pathology and disease heterogeneity. Often mixed amyloid–tau–synuclein pathologies contribute to the clinical spectrum of AD. Commercial blood-based RT-QuIC platforms are being developed, and several biotechnology companies (e.g., Amprion Inc., C2N Diagnostics) already offer CSF testing. These developments hold promise for translating seed amplification-based α-Syn assays from research laboratories into clinical and industrial environments in the near future.

## Conclusion

The pathological aggregation of α-Syn is a key signature of synucleinopathies such as PD, DLB, and MSA. Structural studies have revealed distinct conformers and mutations of α-Syn across diseases, offering mechanistic insight and guiding biomarker development. RT-QuIC has emerged as a powerful tool for detecting misfolded α-Syn with high sensitivity and specificity, even in minute quantities from CSF and peripheral tissues. However, the choice and quality of the recombinant α-Syn substrate remain the most critical factor influencing assay sensitivity, specificity, and reproducibility. Substrate variants, such as familial mutations or truncations, can significantly affect the assay’s kinetics and differentiating power—particularly in distinguishing between PD, DLB, and MSA. While technical parameters like temperature, shaking, and buffer composition also contribute, none are as essential to assay performance as the substrate. To transition RT-QuIC from research to routine clinical diagnostics, it is crucial to optimize substrate quality, standardize protocols across laboratories, and expand its application to non-invasive biospecimens. These efforts will help fulfill RT-QuIC’s potential as a robust, scalable, and disease-specific biomarker platform for early diagnosis and stratification in synucleinopathies.

## Supplementary Information

Below is the link to the electronic supplementary material.

ESM 1 (DOCX 1.04 MB)

## Data Availability

No datasets were generated or analysed during the current study.

## References

[1] Wang L, Das U, Scott DA et al (2014) α-Synuclein multimers cluster synaptic vesicles and attenuate recycling. Curr Biol 24:2319–2326. 10.1016/j.cub.2014.08.027 25264250 10.1016/j.cub.2014.08.027 PMC4190006

[2] Diao J, Burré J, Vivona S et al (2013) Native α-synuclein induces clustering of synaptic-vesicle mimics via binding to phospholipids and synaptobrevin-2/VAMP2. Elife 2:e00592. 10.7554/eLife.00592 23638301 10.7554/eLife.00592 PMC3639508

[3] Fouke KE, Wegman ME, Weber SA et al (2021) Synuclein regulates synaptic vesicle clustering and docking at a vertebrate synapse. Front Cell Dev Biol 9:774650. 10.3389/fcell.2021.774650 34901020 10.3389/fcell.2021.774650 PMC8660973

[4] Burré J, Sharma M, Südhof TC (2014) Α-synuclein assembles into higher-order multimers upon membrane binding to promote SNARE complex formation. Proc Natl Acad Sci U S A 111:E4274–E4283. 10.1073/pnas.1416598111 25246573 10.1073/pnas.1416598111 PMC4210039

[5] Burré J, Sharma M, Tsetsenis T et al (2010) α-Synuclein promotes SNARE-complex assembly *in vivo* and *in vitro*. Science 329:1663–1667. 10.1126/science.1195227 20798282 10.1126/science.1195227 PMC3235365

[6] Sun J, Wang L, Bao H et al (2019) Functional cooperation of α-synuclein and VAMP2 in synaptic vesicle recycling. Proc Natl Acad Sci USA 166:11113–11115. 10.1073/pnas.1903049116 10.1073/pnas.1903049116 PMC6561242 31110017

[7] Scott D, Roy S (2012) Α-synuclein inhibits intersynaptic vesicle mobility and maintains recycling-pool homeostasis. J Neurosci 32:10129–10135. 10.1523/JNEUROSCI.0535-12.2012 22836248 10.1523/JNEUROSCI.0535-12.2012 PMC3426499

[8] Nemani VM, Lu W, Berge V et al (2010) Increased expression of α-synuclein reduces neurotransmitter release by inhibiting synaptic vesicle reclustering after endocytosis. Neuron 65:66–79. 10.1016/j.neuron.2009.12.023 20152114 10.1016/j.neuron.2009.12.023 PMC3119527

[9] Pei Y, Maitta RW (2019) Alpha synuclein in hematopoiesis and immunity. Heliyon 5:e02590. 10.1016/j.heliyon.2019.e02590 31692680 10.1016/j.heliyon.2019.e02590 PMC6806402

[10] Maroteaux L, Campanelli JT, Scheller RH (1988) Synuclein: a neuron-specific protein localized to the nucleus and presynaptic nerve terminal. J Neurosci 8:2804–2815. 10.1523/jneurosci.08-08-02804.1988 3411354 10.1523/JNEUROSCI.08-08-02804.1988 PMC6569395

[11] Li WW, Yang R, Guo JC et al (2007) Localization of α-synuclein to mitochondria within midbrain of mice. NeuroReport 18:1543–1546. 10.1097/WNR.0b013e3282f03db4 17885598 10.1097/WNR.0b013e3282f03db4

[12] Gosavi N, Lee H-J, Lee JS et al (2002) Golgi fragmentation occurs in the cells with prefibrillar α-synuclein aggregates and precedes the formation of fibrillar inclusion. J Biol Chem 277:48984–48992. 10.1074/jbc.M208194200 12351643 10.1074/jbc.M208194200

[13] Hoozemans JJM, van Haastert ES, Eikelenboom P et al (2007) Activation of the unfolded protein response in Parkinson’s disease. Biochem Biophys Res Commun 354:707–711. 10.1016/j.bbrc.2007.01.043 17254549 10.1016/j.bbrc.2007.01.043

[14] Lee HJ, Khoshaghideh F, Patel S, Lee SJ (2004) Clearance of α-synuclein oligomeric intermediates via the lysosomal degradation pathway. J Neurosci 24:1888–1896. 10.1523/JNEUROSCI.3809-03.2004 14985429 10.1523/JNEUROSCI.3809-03.2004 PMC6730405

[15] De Mattos EP, Wentink A, Nussbaum-Krammer C et al (2020) Protein quality control pathways at the crossroad of synucleinopathies. J Parkinsons Dis 10:369–382. 10.3233/JPD-191790 31985474 10.3233/JPD-191790 PMC7242842

[16] Kosaka K, Matsushita M, Oyanagi S, Mehraein P (1980) A cliniconeurophathological study of the “Lewy body disease.” Seishin Shinkeigaku Zasshi 82:292–311 6250177

[17] Tu P, Galvin JE, Baba M et al (1998) Glial cytoplasmic inclusions in white matter oligodendrocytes of multiple system atrophy brains contain insoluble α-synuclein. Ann Neurol 44:415–422 9749615 10.1002/ana.410440324

[18] Papp MI, Lantos PL (1994) The distribution of oligodendroglial inclusions in multiple system atrophy and its relevance to clinical symptomatology. Brain 117:235–243. 10.1093/brain/117.2.235 8186951 10.1093/brain/117.2.235

[19] Wong YC, Krainc D (2017) α-Synuclein toxicity in neurodegeneration: mechanism and therapeutic strategies. Nat Med 23:1–13. 10.1038/nm.4269 28170377 10.1038/nm.4269 PMC8480197

[20] Willis AW, Roberts E, Beck JC et al (2022) Incidence of Parkinson disease in North America. NPJ Parkinsons Dis 8:170. 10.1038/s41531-022-00410-y 36522332 10.1038/s41531-022-00410-y PMC9755252

[21] Kulcsarova K, Skorvanek M, Postuma RB, Berg D (2024) Defining Parkinson’s disease: past and future. J Parkinsons Dis 14(s2):S257–S271. 10.3233/jpd-230411 38489197 10.3233/JPD-230411 PMC11492139

[22] Andersen KB, Krishnamurthy A, Just MK et al (2025) Sympathetic and parasympathetic subtypes of body-first Lewy body disease observed in postmortem tissue from prediagnostic individuals. Nat Neurosci 28:925–936. 10.1038/s41593-025-01910-9 40082617 10.1038/s41593-025-01910-9 PMC12081295

[23] Horsager J, Andersen KB, Knudsen K et al (2020) Brain-first versus body-first Parkinson’s disease: a multimodal imaging case-control study. Brain 143:3077–3088. 10.1093/brain/awaa238 32830221 10.1093/brain/awaa238

[24] Borghammer P, Horsager J, Andersen K et al (2021) Neuropathological evidence of body-first vs. brain-first Lewy body disease. Neurobiol Dis 161:105557. 10.1016/j.nbd.2021.105557 34763110 10.1016/j.nbd.2021.105557

[25] Teixeira D, Cardoso I (2017) Genes involved in the development of Parkinson. Open J Park Dis Treat 1:39–51. 10.17352/ojpdt.000005

[26] Gallagher J, Gochanour C, Caspell-Garcia C et al (2024) Long-term dementia risk in Parkinson disease. Neurology 103:e209699. 10.1212/WNL.0000000000209699 39110916 10.1212/WNL.0000000000209699 PMC11318527

[27] Koros C, Stefanis L, Scarmeas N (2022) Parkinsonism and dementia. J Neurol Sci 433:120015. 10.1016/j.jns.2021.120015 34642023 10.1016/j.jns.2021.120015

[28] Desai U, Chandler J, Kirson N et al (2022) Epidemiology and economic burden of Lewy body dementia in the United States. Curr Med Res Opin 38:1177–1188. 10.1080/03007995.2022.2059978 35442134 10.1080/03007995.2022.2059978

[29] Prasad S, Katta MR, Abhishek S et al (2023) Recent advances in Lewy body dementia: a comprehensive review. Dis-a-Month 69:101441. 10.1016/j.disamonth.2022.101441 10.1016/j.disamonth.2022.101441 35690493

[30] Poewe W, Stankovic I, Halliday G et al (2022) Multiple system atrophy. Nat Rev Dis Primers 8:1–21. 10.1038/s41572-022-00382-6 10.1038/s41572-022-00382-6 36008429

[31] Dorsey ER, Sherer T, Okun MS, Bloemd BR (2018) The emerging evidence of the Parkinson pandemic. J Parkinsons Dis 8:S1–S8. 10.3233/JPD-181474 30584159 10.3233/JPD-181474 PMC6311367

[32] Jensen PH, Nielsen MS, Jakes R et al (1998) Binding of α-synuclein to brain vesicles is abolished by familial Parkinson’s disease mutation. J Biol Chem 273:26292–26294. 10.1074/jbc.273.41.26292 9756856 10.1074/jbc.273.41.26292

[33] Zhao M, Cascio D, Sawaya MR, Eisenberg D (2011) Structures of segments of α-synuclein fused to maltose-binding protein suggest intermediate states during amyloid formation. Protein Sci. 10.1002/pro.630 21462277 10.1002/pro.630 PMC3104229

[34] Goedert M, Griesinger C, Outeiro TF et al (2024) Abandon the NAC in α-synuclein. Lancet Neurol 23:669. 10.1016/S1474-4422(24)00176-5 38876744 10.1016/S1474-4422(24)00176-5

[35] Rodriguez JA, Ivanova MI, Sawaya MR et al (2015) Structure of the toxic core of α-synuclein from invisible crystals. Nature 525:486–490. 10.1038/nature15368 26352473 10.1038/nature15368 PMC4791177

[36] Iwai A, Yoshimoto M, Masliah E, Saitoh T (1995) Non-Aβ component of Alzheimer’s disease amyloid (NAC) is amyloidogenic. Biochemistry 34:10139–10145. 10.1021/bi00032a006 7640267 10.1021/bi00032a006

[37] Giasson BI, Murray IVJ, Trojanowski JQ, Lee VMY (2001) A hydrophobic stretch of 12 amino acid residues in the middle of α-synuclein is essential for filament assembly. J Biol Chem 276:2380–2386. 10.1074/jbc.M008919200 11060312 10.1074/jbc.M008919200

[38] Murray IVJ, Giasson BI, Quinn SM et al (2003) Role of α-synuclein carboxy-terminus on fibril formation in vitro. Biochemistry 42:8530–8540. 10.1021/bi027363r 12859200 10.1021/bi027363r

[39] Nielsen MS, Vorum H, Lindersson E, Jensen PH (2001) Ca2+ binding to α-synuclein regulates ligand binding and oligomerization. J Biol Chem 276:22680–22684. 10.1074/jbc.M101181200 11312271 10.1074/jbc.M101181200

[40] Allison JR, Varnai P, Dobson CM, Vendruscolo M (2009) Determination of the free energy landscape of α-synuclein using spin label nuclear magnetic resonance measurements. J Am Chem Soc 131:67–70. 10.1021/ja904716h 10.1021/ja904716h 20028147

[41] Trexler AJ, Rhoades E (2012) N-terminal acetylation is critical for forming α-helical oligomer of α-synuclein. Protein Sci 21:601–605. 10.1002/pro.2056 22407793 10.1002/pro.2056 PMC3403458

[42] O’Leary EI, Lee JC (2019) Interplay between α-synuclein amyloid formation and membrane structure. Biochim. Biophys. Acta - Proteins Proteomics 1867 10.1016/j.bbapap.2018.09.012 PMC6445794 30287222

[43] Ulmer TS, Bax A, Cole NB, Nussbaum RL (2005) Structure and dynamics of micelle-bound human α-synuclein. J Biol Chem 280:9595–9603. 10.1074/jbc.M411805200 15615727 10.1074/jbc.M411805200

[44] Bartels T, Choi JG, Selkoe DJ (2011) α-Synuclein occurs physiologically as a helically folded tetramer that resists aggregation. Nature 477:107–111. 10.1038/nature10324 21841800 10.1038/nature10324 PMC3166366

[45] Bhattacharya S, Xu L, Arrué L et al (2024) Conformational selection of α-synuclein tetramers at biological interfaces. J Chem Inf Model 64:8010–8023. 10.1021/acs.jcim.4c01459 39377660 10.1021/acs.jcim.4c01459 PMC11523075

[46] Xu L, Bhattacharya S, Thompson D (2018) Re-designing the α-synuclein tetramer. Chem Commun 54:8080–8083. 10.1039/c8cc04054k 10.1039/c8cc04054k 29971274

[47] Jao CC, Hegde BG, Chenb J et al (2008) Structure of membrane-bound α-synuclein from site-directed spin labeling and computational refinement. Proc Natl Acad Sci USA 105:19666–19671. 10.1073/pnas.0807826105 19066219 10.1073/pnas.0807826105 PMC2605001

[48] Xu L, Bhattacharya S, Thompson D (2019) On the ubiquity of helical α-synuclein tetramers. Phys Chem Chem Phys 21:12036–12043. 10.1039/c9cp02464f 31135803 10.1039/c9cp02464f

[49] Fernández RD, Lucas HR (2018) Isolation of recombinant tetrameric N-acetylated α-synuclein. Protein Expr Purif 152:146–154. 10.1016/j.pep.2018.07.008 30041032 10.1016/j.pep.2018.07.008

[50] Dettmer U, Newman AJ, von Saucken VE et al (2015) KTKEGV repeat motifs are key mediators of normal α-synuclein tetramerization: their mutation causes excess monomers and neurotoxicity. Proc Natl Acad Sci U S A 112:9596–9601. 10.1073/pnas.1505953112 26153422 10.1073/pnas.1505953112 PMC4534262

[51] Dettmer U, Newman AJ, Soldner F et al (2015) Parkinson-causing α-synuclein missense mutations shift native tetramers to monomers as a mechanism for disease initiation. Nat Commun 6:7314. 10.1038/ncomms8314 26076669 10.1038/ncomms8314 PMC4490410

[52] Nuber S, Rajsombath M, Minakaki G et al (2018) Abrogating native α-synuclein tetramers in mice causes a L-DOPA-responsive motor syndrome closely resembling Parkinson’s disease. Neuron 100:75-90.e5. 10.1016/j.neuron.2018.09.014 30308173 10.1016/j.neuron.2018.09.014 PMC6211795

[53] Wang W, Perovic I, Chittuluru J et al (2011) A soluble α-synuclein construct forms a dynamic tetramer. Proc Natl Acad Sci USA 108:17797–17802. 10.1073/pnas.1113260108 22006323 10.1073/pnas.1113260108 PMC3203798

[54] de Boni L, Wallis A, Hays Watson A et al (2024) Aggregation-resistant alpha-synuclein tetramers are reduced in the blood of Parkinson’s patients. EMBO Mol Med 16:1657–1674. 10.1038/s44321-024-00083-5 38839930 10.1038/s44321-024-00083-5 PMC11250827

[55] Emin D, Zhang YP, Lobanova E et al (2022) Small soluble α-synuclein aggregates are the toxic species in Parkinson’s disease. Nat Commun 13:5512. 10.1038/s41467-022-33252-6 36127374 10.1038/s41467-022-33252-6 PMC9489799

[56] Chen SW, Drakulic S, Deas E et al (2015) Structural characterization of toxic oligomers that are kinetically trapped during α-synuclein fibril formation. Proc Natl Acad Sci U S A 112:E1994–E2003. 10.1073/pnas.1421204112 25855634 10.1073/pnas.1421204112 PMC4413268

[57] Cremades N, Cohen SIA, Deas E et al (2012) Direct observation of the interconversion of normal and toxic forms of α-synuclein. Cell 149:1048–1059. 10.1016/j.cell.2012.03.037 22632969 10.1016/j.cell.2012.03.037 PMC3383996

[58] Fusco G, Chen SW, Williamson PTF et al (2017) Structural basis of membrane disruption and cellular toxicity by a-synuclein oligomers. Science (80-) 358:1440–1443. 10.1126/science.aan6160 10.1126/science.aan6160 29242346

[59] van Diggelen F, Tepper AWJW, Apetri MM, Otzen DE (2017) α-Synuclein oligomers: a study in diversity. Isr J Chem 57:2037–2054. 10.1002/ijch.201600116

[60] Santos J, Cuellar J, Pallarès I et al (2024) A targetable N-terminal motif orchestrates α-synuclein oligomer-to-fibril conversion. J Am Chem Soc 146:12702–12711. 10.1021/jacs.4c02262 38683963 10.1021/jacs.4c02262 PMC11082882

[61] Lau HHC, Martinez-Valbuena I, So RWL et al (2023) The G51D SNCA mutation generates a slowly progressive α-synuclein strain in early-onset Parkinson’s disease. Acta Neuropathol Commun 11:72. 10.1186/s40478-023-01570-5 37138318 10.1186/s40478-023-01570-5 PMC10155462

[62] Sant V, Matthes D, Mazal H et al (2025) Lipidic folding pathway of α-synuclein via a toxic oligomer. Nat Commun 16:760. 10.1038/s41467-025-55849-3 39824800 10.1038/s41467-025-55849-3 PMC11742675

[63] Schwarz TC, Beier A, Ledolter K et al (2023) High-resolution structural information of membrane-bound α-synuclein provides insight into the MoA of the anti-Parkinson drug UCB0599. Proc Natl Acad Sci U S A 120:e2201910120. 10.1073/pnas.2201910120 37027427 10.1073/pnas.2201910120 PMC10104497

[64] Xu CK, Meisl G, Andrzejewska EA et al (2024) α-Synuclein oligomers form by secondary nucleation. Nat Commun 15:7083. 10.1038/s41467-024-50692-4 39153989 10.1038/s41467-024-50692-4 PMC11330488

[65] Blömeke L, Pils M, Kraemer-Schulien V et al (2022) Quantitative detection of α-synuclein and tau oligomers and other aggregates by digital single particle counting. NPJ Parkinsons Dis 8:68. 10.1038/s41531-022-00330-x 35655068 10.1038/s41531-022-00330-x PMC9163356

[66] Danzer KM, Haasen D, Karow AR et al (2007) Different species of α-synuclein oligomers induce calcium influx and seeding. J Neurosci 27:9220–9232. 10.1523/JNEUROSCI.2617-07.2007 17715357 10.1523/JNEUROSCI.2617-07.2007 PMC6672196

[67] Rey NL, Petit GH, Bousset L et al (2013) Transfer of human α-synuclein from the olfactory bulb to interconnected brain regions in mice. Acta Neuropathol 126:555–573. 10.1007/s00401-013-1160-3 23925565 10.1007/s00401-013-1160-3 PMC3789892

[68] Kumari P, Ghosh D, Vanas A et al (2021) Structural insights into α-synuclein monomer–fibril interactions. Proc Natl Acad Sci U S A 118:e2012171118. 10.1073/pnas.2012171118 33649211 10.1073/pnas.2012171118 PMC7958257

[69] Yang Y, Shi Y, Schweighauser M et al (2022) Structures of α-synuclein filaments from human brains with Lewy pathology. Nature 610:791–795. 10.1038/s41586-022-05319-3 36108674 10.1038/s41586-022-05319-3 PMC7613749

[70] Schweighauser M, Shi Y, Tarutani A et al (2020) Structures of α-synuclein filaments from multiple system atrophy. Nature 585:464–469. 10.1038/s41586-020-2317-6 32461689 10.1038/s41586-020-2317-6 PMC7116528

[71] Yan NL, Candido F, Tse E et al (2024) Cryo-EM structure of a novel α-synuclein filament subtype from multiple system atrophy. FEBS Lett 599:33–40. 10.1002/1873-3468.15048 39511911 10.1002/1873-3468.15048 PMC11726156

[72] Yang Y, Garringer HJ, Shi Y et al (2023) New SNCA mutation and structures of α-synuclein filaments from juvenile-onset synucleinopathy. Acta Neuropathol 145:561–572. 10.1007/s00401-023-02550-8 36847833 10.1007/s00401-023-02550-8 PMC10119069

[73] Dhavale DD, Barclay AM, Borcik CG et al (2024) Structure of alpha-synuclein fibrils derived from human Lewy body dementia tissue. Nat Commun 15:2750. 10.1038/s41467-024-46832-5 38553463 10.1038/s41467-024-46832-5 PMC10980826

[74] Guerrero-Ferreira R, Taylor NMI, Arteni AA et al (2019) Two new polymorphic structures of human full-length alpha-synuclein fibrils solved by cryo-electron microscopy. Elife 8:e48907. 10.7554/eLife.48907 31815671 10.7554/eLife.48907 PMC6957273

[75] Li B, Ge P, Murray KA et al (2018) Cryo-EM of full-length α-synuclein reveals fibril polymorphs with a common structural kernel. Nat Commun 9:3609. 10.1038/s41467-018-05971-2 30190461 10.1038/s41467-018-05971-2 PMC6127345

[76] Lövestam S, Schweighauser M, Matsubara T et al (2021) Seeded assembly *in vitro* does not replicate the structures of α-synuclein filaments from multiple system atrophy. FEBS Open Bio 11:999–1013. 10.1002/2211-5463.13110 33548114 10.1002/2211-5463.13110 PMC8016116

[77] Boyer DR, Li B, Sun C et al (2020) The α-synuclein hereditary mutation E46K unlocks a more stable, pathogenic fibril structure. Proc Natl Acad Sci USA 117:3592–3602. 10.1073/pnas.1917914117 32015135 10.1073/pnas.1917914117 PMC7035510

[78] Frey L, Ghosh D, Qureshi BM et al (2024) On the pH-dependence of α-synuclein amyloid polymorphism and the role of secondary nucleation in seed-based amyloid propagation. Elife 12:RP93562. 10.7554/elife.93562.4 39196271 10.7554/eLife.93562 PMC11357353

[79] Krüger R, Kuhn W, Müller T et al (1998) Ala30Pro mutation in the gene encoding α-synuclein in Parkinson’s disease. Nat Genet 18:106–108. 10.1038/ng0298-106 9462735 10.1038/ng0298-106

[80] Zarranz JJ, Alegre J, Gómez-Esteban JC et al (2004) The new mutation, E46K, of α-synuclein causes Parkinson and Lewy body dementia. Ann Neurol 55:164–173. 10.1002/ana.10795 14755719 10.1002/ana.10795

[81] Proukakis C, Dudzik CG, Brier T et al (2013) A novel α-synuclein missense mutation in Parkinson disease. Neurology 80:1062–1064. 10.1212/WNL.0b013e31828727ba 23427326 10.1212/WNL.0b013e31828727ba PMC3653201

[82] Appel-Cresswell S, Vilarino-Guell C, Encarnacion M et al (2013) Alpha-synuclein p. H50Q, a novel pathogenic mutation for Parkinson’s disease. Mov Disord 28:811–813. 10.1002/mds.25421 23457019 10.1002/mds.25421

[83] Lesage S, Anheim M, Letournel F et al (2013) G51D α-synuclein mutation causes a novel Parkinsonian-pyramidal syndrome. Ann Neurol 73:459–471. 10.1002/ana.23894 23526723 10.1002/ana.23894

[84] Polymeropoulos MH, Lavedan C, Leroy E et al (1997) Mutation in the α-synuclein gene identified in families with Parkinson’s disease. Science 276:2045–2047. 10.1126/science.276.5321.2045 9197268 10.1126/science.276.5321.2045

[85] Yoshino H, Hirano M, Stoessl AJ et al (2017) Homozygous alpha-synuclein p. A53V in familial Parkinson’s disease. Neurobiol Aging 57:248.e7-248.e12. 10.1016/j.neurobiolaging.2017.05.022 28666710 10.1016/j.neurobiolaging.2017.05.022

[86] Picillo M, Lizarraga KJ, Friesen EL et al (2018) Parkinsonism due to A53E α-synuclein gene mutation: clinical, genetic, epigenetic, and biochemical features. Mov Disord 33:1950–1955. 10.1002/mds.27506 30423204 10.1002/mds.27506

[87] Scholz SW, Houlden H, Schulte C et al (2009) SNCA variants are associated with increased risk for multiple system atrophy. Ann Neurol 65:610–614. 10.1002/ana.21685 19475667 10.1002/ana.21685 PMC3520128

[88] Al-Chalabi A, Dürr A, Wood NW et al (2009) Genetic variants of the α-synuclein gene SNCA are associated with multiple system atrophy. PLoS ONE 4:e7114. 10.1371/journal.pone.0007114 19771175 10.1371/journal.pone.0007114 PMC2743996

[89] Guella I, Evans DM, Szu-Tu C et al (2016) α-Synuclein genetic variability: a biomarker for dementia in Parkinson disease. Ann Neurol 79:991–999. 10.1002/ana.24664 27091628 10.1002/ana.24664

[90] Zhao K, Li Y, Liu Z et al (2020) Parkinson’s disease associated mutation E46K of α-synuclein triggers the formation of a distinct fibril structure. Nat Commun 11:2643. 10.1038/s41467-020-16386-3 32457390 10.1038/s41467-020-16386-3 PMC7250837

[91] Long H, Zheng W, Liu Y et al (2021) Wild-type α-synuclein inherits the structure and exacerbated neuropathology of E46K mutant fibril strain by cross-seeding. Proc Natl Acad Sci USA 118:e2012435118. 10.1073/pnas.2012435118 33972418 10.1073/pnas.2012435118 PMC8158012

[92] Brücke C, Al-Azzani M, Ramalingam N et al (2025) A novel alpha-synuclein G14R missense variant is associated with atypical neuropathological features. medRxiv [Preprint] 10.1101/2024.09.23.24313864 10.1186/s13024-025-00889-y PMC12465293 41013605

[93] Boyer DR, Li B, Sun C et al (2019) Structures of fibrils formed by α-synuclein hereditary disease mutant H50Q reveal new polymorphs. Nat Struct Mol Biol 26:1044–1052. 10.1038/s41594-019-0322-y 31695184 10.1038/s41594-019-0322-y PMC6907165

[94] Sun Y, Long H, Xia W et al (2021) The hereditary mutation G51D unlocks a distinct fibril strain transmissible to wild-type α-synuclein. Nat Commun 12:6252. 10.1038/s41467-021-26433-2 34716315 10.1038/s41467-021-26433-2 PMC8556266

[95] Sun Y, Hou S, Zhao K et al (2020) Cryo-EM structure of full-length α-synuclein amyloid fibril with Parkinson’s disease familial A53T mutation. Cell Res 30:360–362. 10.1038/s41422-020-0299-4 32203130 10.1038/s41422-020-0299-4 PMC7118165

[96] Sun C, Zhou K, DePaola P et al (2023) Cryo-EM structure of amyloid fibril formed by α-synuclein hereditary A53E mutation reveals a distinct protofilament interface. J Biol Chem 299:104566. 10.1016/j.jbc.2023.104566 36871760 10.1016/j.jbc.2023.104566 PMC10124909

[97] Hassanzadeh K, Liu J, Maddila S, Mouradian MM (2024) Posttranslational modifications of α-synuclein, their therapeutic potential, and crosstalk in health and neurodegenerative diseases. Pharmacol Rev 76:1254–1290. 10.1124/pharmrev.123.001111 39164116 10.1124/pharmrev.123.001111 PMC11549938

[98] Anderson JP, Walker DE, Goldstein JM et al (2006) Phosphorylation of Ser-129 is the dominant pathological modification of α-synuclein in familial and sporadic Lewy body disease. J Biol Chem 281:29739–29752. 10.1074/jbc.M600933200 16847063 10.1074/jbc.M600933200

[99] Fujiwara H, Hasegawa M, Dohmae N et al (2002) Α-synuclein is phosphorylated in synucleinopathy lesions. Nat Cell Biol 4:160–164. 10.1038/ncb748 11813001 10.1038/ncb748

[100] Ghanem SS, Majbour NK, Vaikath NN et al (2022) Α-synuclein phosphorylation at serine 129 occurs after initial protein deposition and inhibits seeded fibril formation and toxicity. Proc Natl Acad Sci U S A 119:e2109617119. 10.1073/pnas.2109617119 35353605 10.1073/pnas.2109617119 PMC9169642

[101] Zhao K, Lim YJ, Liu Z et al (2020) Parkinson’s disease-related phosphorylation at Tyr39 rearranges α-synuclein amyloid fibril structure revealed by cryo-EM. Proc Natl Acad Sci U S A 117:20305–20315. 10.1073/PNAS.1922741117 32737160 10.1073/pnas.1922741117 PMC7443891

[102] Hu J, Xia W, Zeng S et al (2024) Phosphorylation and O-GlcNAcylation at the same α-synuclein site generate distinct fibril structures. Nat Commun 15:2677. 10.1038/s41467-024-46898-1 38538591 10.1038/s41467-024-46898-1 PMC10973503

[103] Balana AT, Mahul-Mellier AL, Nguyen BA et al (2024) O-GlcNAc forces an α-synuclein amyloid strain with notably diminished seeding and pathology. Nat Chem Biol 20:645–655. 10.1038/s41589-024-01551-2 10.1038/s41589-024-01551-2 PMC11062923 38347213

[104] Zenko D, Marsh J, Castle AR et al (2023) Monitoring α-synuclein ubiquitination dynamics reveals key endosomal effectors mediating its trafficking and degradation. Sci Adv 9:eadd8910. 10.1126/sciadv.add8910 37315142 10.1126/sciadv.add8910 PMC10266730

[105] Duda JE, Giasson BI, Chen Q et al (2000) Widespread nitration of pathological inclusions in neurodegenerative synucleinopathies. Am J Pathol 157:1439–1445. 10.1016/S0002-9440(10)64781-5 11073803 10.1016/S0002-9440(10)64781-5 PMC1885725

[106] Giasson BI, Duda JE, Murray IVJ et al (2000) Oxidative damage linked to neurodegeneration by selective α-synuclein nitration in synucleinopathy lesions. Science (80-) 290:985–989. 10.1126/science.290.5493.985 10.1126/science.290.5493.985 11062131

[107] Danielson SR, Held JM, Schilling B et al (2009) Preferentially increased nitration of α-synuclein at tyrosine-39 in a cellular oxidative model of Parkinson’s disease. Anal Chem 81:7823–7828. 10.1021/ac901176t 19697948 10.1021/ac901176t PMC2748813

[108] Carmo-Gonçalves P, Pinheiro AS, Romão L et al (2014) UV-induced selective oxidation of Met5 to Met-sulfoxide leads to the formation of neurotoxic fibril-incompetent α-synuclein oligomers. Amyloid 21:163–174. 10.3109/13506129.2014.912208 24784227 10.3109/13506129.2014.912208

[109] Wang X, Liang T, Jin A et al (2025) C-terminal radical oxidation inhibits α-synuclein aggregation and cytotoxicity via an oxidative oligomer-disrupting pathway. J Am Chem Soc 147:23136–23144. 10.1021/jacs.5c06792 40530874 10.1021/jacs.5c06792

[110] Prasad K, Beach TG, Hedreen J, Richfield EK (2012) Critical role of truncated α-synuclein and aggregates in Parkinson’s disease and incidental Lewy body disease. Brain Pathol 22:811–825. 10.1111/j.1750-3639.2012.00597.x 22452578 10.1111/j.1750-3639.2012.00597.x PMC5505643

[111] Li W, West N, Colla E et al (2005) Aggregation promoting C-terminal truncation of α-synuclein is a normal cellular process and is enhanced by the familial Parkinson’s disease-linked mutations. Proc Natl Acad Sci USA 102:2162–2167. 10.1073/pnas.0406976102 15684072 10.1073/pnas.0406976102 PMC548541

[112] Sorrentino ZA, Vijayaraghavan N, Gorion KM et al (2018) Physiological C-terminal truncation of -synuclein potentiates the prion-like formation of pathological inclusions. J Biol Chem 293:18914–18932. 10.1074/jbc.RA118.005603 30327435 10.1074/jbc.RA118.005603 PMC6295729

[113] Van Der Wateren IM, Knowles TPJ, Buell AK et al (2018) C-terminal truncation of α-synuclein promotes amyloid fibril amplification at physiological pH. Chem Sci 9:5506–5516. 10.1039/c8sc01109e 30061982 10.1039/c8sc01109e PMC6048717

[114] Farzadfard A, Pedersen JN, Meisl G et al (2022) The C-terminal tail of α-synuclein protects against aggregate replication but is critical for oligomerization. Commun Biol 5:123. 10.1038/s42003-022-03059-8 35145226 10.1038/s42003-022-03059-8 PMC8831632

[115] Lewis KA, Su Y, Jou O et al (2010) Abnormal neurites containing C-terminally truncated α-synuclein are present in Alzheimer’s disease without conventional Lewy body pathology. Am J Pathol 177:3037–3050. 10.2353/ajpath.2010.100552 21056999 10.2353/ajpath.2010.100552 PMC2993276

[116] Lewis KA, Yaeger A, Demartino GN, Thomas PJ (2010) Accelerated formation of α-synuclein oligomers by concerted action of the 20s proteasome and familial Parkinson mutations. J Bioenerg Biomembr 42:85–95. 10.1007/s10863-009-9258-y 20148295 10.1007/s10863-009-9258-y PMC3266686

[117] Wang W, Nguyen LTT, Burlak C et al (2016) Caspase-1 causes truncation and aggregation of the Parkinson’s disease-associated protein α-synuclein. Proc Natl Acad Sci U S A 113:9587–9592. 10.1073/pnas.1610099113 27482083 10.1073/pnas.1610099113 PMC5003239

[118] Ni X, McGlinchey RP, Jiang J, Lee JC (2019) Structural insights into α-synuclein fibril polymorphism: effects of Parkinson’s disease-related C-terminal truncations. J Mol Biol 431:3913–3919. 10.1016/j.jmb.2019.07.001 31295458 10.1016/j.jmb.2019.07.001 PMC6733637

[119] McGlinchey RP, Ni X, Shadish JA et al (2021) The N terminus of α-synuclein dictates fibril formation. Proc Natl Acad Sci U S A 118:e2023487118. 10.1073/pnas.2023487118 34452994 10.1073/pnas.2023487118 PMC8536336

[120] Dewison KM, Rowlinson B, Machin JM et al (2024) Residues 2 to 7 of α- synuclein regulate amyloid formation via lipid- dependent and lipid- independent pathways. Proc Natl Acad Sci 121:e2315006121. 10.1073/pnas 39133842 10.1073/pnas.2315006121 PMC11348338

[121] Mollenhauer B (2023) Status of current biofluid biomarkers in Parkinson’s disease. Mov Disord Clin Pract 10:S18–S20. 10.1002/mdc3.13753 37637982 10.1002/mdc3.13753 PMC10448129

[122] Chopra A, Lang AE, Höglinger G, Outeiro TF (2024) Towards a biological diagnosis of PD. Park Relat Disord 122:106078. 10.1016/j.parkreldis.2024.106078 10.1016/j.parkreldis.2024.106078 38472075

[123] Mammana A, Baiardi S, Rossi M et al (2024) Improving protocols for α-synuclein seed amplification assays: analysis of preanalytical and analytical variables and identification of candidate parameters for seed quantification. Clin Chem Lab Med 62(10):2001–2010. 10.1515/cclm-2023-1472 38456740 10.1515/cclm-2023-1472

[124] Stoker TB, Greenland JC (2018) Parkinson’s disease: pathogenesis and clinical aspects. Exon Publications, Brisbane. 10.15586/codonpublications.parkinsonsdisease.2018 30702835

[125] Sano K, Satoh K, Atarashi R et al (2013) Early detection of abnormal prion protein in genetic human prion diseases now possible using real-time QuIC assay. PLoS ONE 8:e54915. 10.1371/journal.pone.0054915 23372790 10.1371/journal.pone.0054915 PMC3556051

[126] Atarashi R, Satoh K, Sano K et al (2011) Ultrasensitive human prion detection in cerebrospinal fluid by real-time quaking-induced conversion. Nat Med 17:175–178. 10.1038/nm.2294 21278748 10.1038/nm.2294

[127] Wilham JM, Orrú CD, Bessen RA et al (2010) Rapid end-point quantitation of prion seeding activity with sensitivity comparable to bioassays. PLoS Pathog 6:e1001217. 10.1371/journal.ppat.1001217 21152012 10.1371/journal.ppat.1001217 PMC2996325

[128] van Rumund A, Green AJE, Fairfoul G et al (2019) α-Synuclein real-time quaking-induced conversion in the cerebrospinal fluid of uncertain cases of parkinsonism. Ann Neurol 85:777–781. 10.1002/ana.25447 30801759 10.1002/ana.25447 PMC6593725

[129] Fairfoul G, McGuire LI, Pal S et al (2016) Alpha-synuclein RT-QuIC in the CSF of patients with alpha-synucleinopathies. Ann Clin Transl Neurol 3:812–818. 10.1002/acn3.338 27752516 10.1002/acn3.338 PMC5048391

[130] Groveman BR, Orrù CD, Hughson AG et al (2018) Rapid and ultra-sensitive quantitation of disease-associated α-synuclein seeds in brain and cerebrospinal fluid by αSyn RT-QuIC. Acta Neuropathol Commun 6:7. 10.1186/s40478-018-0508-2 29422107 10.1186/s40478-018-0508-2 PMC5806364

[131] Bongianni M, Ladogana A, Capaldi S et al (2019) α-Synuclein RT-QuIC assay in cerebrospinal fluid of patients with dementia with Lewy bodies. Ann Clin Transl Neurol 6:2120–2126. 10.1002/acn3.50897 31599499 10.1002/acn3.50897 PMC6801172

[132] Peña-Bautista C, Kumar R, Baquero M et al (2023) Misfolded alpha-synuclein detection by RT-QuIC in dementia with Lewy bodies: a systematic review and meta-analysis. Front Mol Biosci 10:1193458. 10.3389/fmolb.2023.1193458 37266333 10.3389/fmolb.2023.1193458 PMC10229818

[133] Levine H (1993) Thioflavine T interaction with synthetic Alzheimer’s disease β-amyloid peptides: detection of amyloid aggregation in solution. Protein Sci. 10.1002/pro.5560020312 8453378 10.1002/pro.5560020312 PMC2142377

[134] Grossauer A, Hemicker G, Krismer F et al (2023) α-Synuclein seed amplification assays in the diagnosis of synucleinopathies using cerebrospinal fluid—a systematic review and meta-analysis. Mov Disord Clin Pract 10:737–774. 10.1002/mdc3.13710 37205253 10.1002/mdc3.13710 PMC10187020

[135] Wang Y, Hu J, Chen X et al (2022) Real-time quaking-induced conversion assay is accurate for Lewy body diseases: a meta-analysis. Neurol Sci 43:4125–4132. 10.1007/s10072-022-06014-x 35312879 10.1007/s10072-022-06014-x

[136] Woerman AL, Kazmi SA, Patel S et al (2018) MSA prions exhibit remarkable stability and resistance to inactivation. Acta Neuropathol 135:49–63. 10.1007/s00401-017-1762-2 28849371 10.1007/s00401-017-1762-2 PMC5756500

[137] Ayers JI, Lee J, Monteiro O et al (2022) Different α-synuclein prion strains cause dementia with Lewy bodies and multiple system atrophy. Proc Natl Acad Sci USA 119:e2113489119. 10.1073/pnas.2113489119 35115402 10.1073/pnas.2113489119 PMC8833220

[138] Porcari R, Proukakis C, Waudby CA et al (2015) The H50Q mutation induces a 10-fold decrease in the solubility of α-synuclein. J Biol Chem 290:2395–2404. 10.1074/jbc.M114.610527 25505181 10.1074/jbc.M114.610527 PMC4303689

[139] Srivastava A, Wang Q, Orru CD et al (2024) Enhanced quantitation of pathological α-synuclein in patient biospecimens by RT-QuIC seed amplification assays. 20:e1012554. 10.1371/journal.ppat.1012554 10.1371/journal.ppat.1012554 PMC11451978 39302978

[140] Wiseman JA, Turner CP, Faull RLM et al (2025) Refining α-synuclein seed amplification assays to distinguish Parkinson’s disease from multiple system atrophy. Transl Neurodegener 14:7. 10.1186/s40035-025-00469-6 39920796 10.1186/s40035-025-00469-6 PMC11804046

[141] Iranzo A, Fairfoul G, Ayudhaya ACN et al (2021) Detection of α-synuclein in CSF by RT-QuIC in patients with isolated rapid-eye-movement sleep behaviour disorder: a longitudinal observational study. Lancet Neurol 20:203–212. 10.1016/S1474-4422(20)30449-X 33609478 10.1016/S1474-4422(20)30449-X

[142] Concha-Marambio L, Weber S, Farris MC et al (2023) Accurate detection of α-synuclein seeds in CSF from iRBD and Parkinson patients in the DeNoPa cohort. 38:567–578. 10.1002/mds.29329.Accurate 10.1002/mds.29329 PMC10153075 36781413

[143] Stefani A, Iranzo A, Holzknecht E et al (2021) Alpha-synuclein seeds in olfactory mucosa of patients with isolated REM sleep behaviour disorder. Brain 144:1118–1126. 10.1093/brain/awab005 33855335 10.1093/brain/awab005

[144] Iranzo A, Mammana A, Muñoz-lopetegi A et al (2023) Misfolded α-synuclein assessment in the skin and CSF by RT-QuIC in isolated REM sleep behavior disorder file:///C:/Users/Nadula/Desktop/NiU work/Papers/Detection/RT-QuIC/2022–2025/Concha-Marambio et al., 2023 Accurate detection of α-Synuclein seeds in CSF. 100:e1944–e1954. 10.1212/WNL.0000000000207147 10.1212/WNL.0000000000207147 PMC10159765 36931726

[145] Rossi M, Candelise N, Baiardi S et al (2020) Ultrasensitive RT-QuIC assay with high sensitivity and specificity for Lewy body-associated synucleinopathies. Acta Neuropathol 140:49–62. 10.1007/s00401-020-02160-8 32342188 10.1007/s00401-020-02160-8 PMC7299922

[146] Candelise N, Schmitz M, Thüne K et al (2020) Effect of the micro-environment on α-synuclein conversion and implication in seeded conversion assays. Transl Neurodegener 9:5 31988747 10.1186/s40035-019-0181-9 PMC6966864

[147] Huang J, Yuan X, Chen L et al (2024) Pathological α-synuclein detected by real-time quaking-induced conversion in synucleinopathies. Exp Gerontol 187:112366. 10.1016/j.exger.2024.112366 38280659 10.1016/j.exger.2024.112366

[148] Kuzkina A, Bargar C, Schmitt D et al (2021) Diagnostic value of skin RT-QuIC in Parkinson’s disease: a two-laboratory study. NPJ Parkinsons Dis 7:99. 10.1038/s41531-021-00242-2 34782640 10.1038/s41531-021-00242-2 PMC8593128

[149] Gunzler S, Tatsuoka C, Elkasaby M et al (2022) Real time quaking induced conversion (RT-QuIC) for skin alpha-synuclein seeding activity in Parkinson disease versus controls (P1–1.Virtual). Neurology. 10.1212/wnl.98.18_supplement.1907

[150] Kluge A, Bunk J, Schaeffer E et al (2022) Detection of neuron-derived pathological α-synuclein in blood. Brain 145:3058–3071. 10.1093/brain/awac115 35722765 10.1093/brain/awac115

[151] Schaeffer E, Kluge A, Schulte C et al (2024) Association of misfolded α-synuclein derived from neuronal exosomes in blood with Parkinson’s disease diagnosis and duration. J Parkinsons Dis 14:667–679. 10.3233/JPD-230390 38669557 10.3233/JPD-230390 PMC11191501

[152] Bsoul R, McWilliam OH, Waldemar G et al (2025) Accurate detection of pathologic α-synuclein in CSF, skin, olfactory mucosa, and urine with a uniform seeding amplification assay. Acta Neuropathol Commun 13:113. 10.1186/s40478-025-02034-8 40413531 10.1186/s40478-025-02034-8 PMC12102825

[153] Orrú CD, Hughson AG, Groveman BR et al (2016) Factors that improve RT-QuIC detection of prion seeding activity. Viruses 8:140. 10.3390/v8050140 27223300 10.3390/v8050140 PMC4885095

[154] Orrú CD, Groveman BR, Hughson AG et al (2015) Rapid and sensitive RT-QuIC detection of human Creutzfeldt-Jakob disease using cerebrospinal fluid. MBio 6:e02451–14. 10.1128/mBio.02451-14 25604790 10.1128/mBio.02451-14 PMC4313917

[155] Parveen S, Alam P, Orrù CD et al (2025) A same day α-synuclein RT-QuIC seed amplification assay for synucleinopathy biospecimens. npj Biosensing 2:1–9. 10.1038/s44328-024-00023-w 10.1038/s44328-024-00023-w PMC11813799 39950033

[156] Han JY, Jang HS, Green AJE, Choi YP (2020) RT-QuIC-based detection of alpha-synuclein seeding activity in brains of dementia with Lewy body patients and of a transgenic mouse model of synucleinopathy. Prion 14:88–94. 10.1080/19336896.2020.1724608 32041499 10.1080/19336896.2020.1724608 PMC7039666

[157] Bargar C, Wang W, Gunzler SA et al (2021) Streamlined alpha-synuclein RT-QuIC assay for various biospecimens in Parkinson’s disease and dementia with Lewy bodies. Acta Neuropathol Commun 9:62. 10.1186/s40478-021-01175-w 33827706 10.1186/s40478-021-01175-w PMC8028088

[158] Quadalti C, Palmqvist S, Hall S et al (2023) Clinical effects of Lewy body pathology in cognitively impaired individuals. Nat Med 29:1964–1970. 10.1038/s41591-023-02449-7 37464058 10.1038/s41591-023-02449-7 PMC10427416

[159] Garrido A, Fairfoul G, Tolosa ES et al (2019) α-Synuclein RT-QuIC in cerebrospinal fluid of LRRK2-linked Parkinson’s disease. Ann Clin Transl Neurol 6:1024–1103. 10.1002/acn3.772 31211166 10.1002/acn3.772 PMC6562027

[160] Sakurai A, Tsunemi T, Ishiguro Y et al (2022) Comorbid alpha synucleinopathies in idiopathic normal pressure hydrocephalus. J Neurol 269:2022–2029. 10.1007/s00415-021-10778-1 34468800 10.1007/s00415-021-10778-1

[161] Shahnawaz M, Tokuda T, Waraga M et al (2017) Development of a biochemical diagnosis of Parkinson disease by detection of α-synuclein misfolded aggregates in cerebrospinal fluid. JAMA Neurol 74:163–172. 10.1001/jamaneurol.2016.4547 27918765 10.1001/jamaneurol.2016.4547

[162] Poggiolini I, Gupta V, Lawton M et al (2022) Diagnostic value of cerebrospinal fluid alpha-synuclein seed quantification in synucleinopathies. Brain 145:584–595. 10.1093/brain/awab431 34894214 10.1093/brain/awab431 PMC9014737

[163] Poggiolini I, Erskine D, Vaikath NN et al (2021) Rt-quic using C-terminally truncated α-synuclein forms detects differences in seeding propensity of different brain regions from synucleinopathies. Biomolecules 11:820. 10.3390/biom11060820 34072869 10.3390/biom11060820 PMC8226794

[164] Cohlberg JA, Li J, Uversky VN, Fink AL (2002) Heparin and other glycosaminoglycans stimulate the formation of amyloid fibrils from α-synuclein in vitro. Biochemistry 41:1502–1511. 10.1021/bi011711s 11814343 10.1021/bi011711s

[165] Atarod D, Mamashli F, Ghasemi A et al (2022) Bivalent metal ions induce formation of α-synuclein fibril polymorphs with different cytotoxicities. Sci Rep 12:11898. 10.1038/s41598-022-15472-4 35831343 10.1038/s41598-022-15472-4 PMC9279330

[166] Lorentzon E, Kumar R, Horvath I, Wittung-Stafshede P (2020) Differential effects of Cu2+ and Fe3+ ions on in vitro amyloid formation of biologically-relevant α-synuclein variants. Biometals 33:97–106. 10.1007/s10534-020-00234-4 32170541 10.1007/s10534-020-00234-4 PMC7295844

[167] Prusiner SB, Woerman AL, Mordes DA et al (2015) Evidence for α-synuclein prions causing multiple system atrophy in humans with parkinsonism. Proc Natl Acad Sci U S A 112:E5308–E5317. 10.1073/pnas.1514475112 26324905 10.1073/pnas.1514475112 PMC4586853

[168] Watts JC, Giles K, Oehler A et al (2013) Transmission of multiple system atrophy prions to transgenic mice. Proc Natl Acad Sci U S A 110:19555–19560. 10.1073/pnas.1318268110 24218576 10.1073/pnas.1318268110 PMC3845125

[169] Koo HJ, Lee HJ, Im H (2008) Sequence determinants regulating fibrillation of human α-synuclein. Biochem Biophys Res Commun 368:772–778. 10.1016/j.bbrc.2008.01.140 18261982 10.1016/j.bbrc.2008.01.140

[170] Khalaf O, Fauvet B, Oueslati A et al (2014) The H50Q mutation enhances α-synuclein aggregation, secretion, and toxicity. J Biol Chem 289:21856–21876. 10.1074/jbc.M114.553297 24936070 10.1074/jbc.M114.553297 PMC4139205

[171] Rutherford NJ, Moore BD, Golde TE, Giasson BI (2014) Divergent effects of the H50Q and G51D SNCA mutations on the aggregation of α-synuclein. J Neurochem 131:859–867. 10.1111/jnc.12806 24984882 10.1111/jnc.12806

[172] Gilboa T, Swank Z, Thakur R et al (2024) Toward the quantification of α-synuclein aggregates with digital seed amplification assays. Proc Natl Acad Sci U S A 121:e2312031121. 10.1073/pnas.2312031121 38194461 10.1073/pnas.2312031121 PMC10801878

