# Supplementary material for: When Proteins Go MAD—Misfolded, Amplified, Detected: Advances in α-Synuclein Pathophysiology and RT-QuIC Detection
Source: Mol Neurobiol. 2026 Jan 9;63(1):352. doi: 10.1007/s12035-025-05600-2 (PMC12789148; doi:10.1007/s12035-025-05600-2)
Supplement: Supplementary file 1 — (DOCX 1.04 MB) [file 12035_2025_5600_MOESM1_ESM.docx]

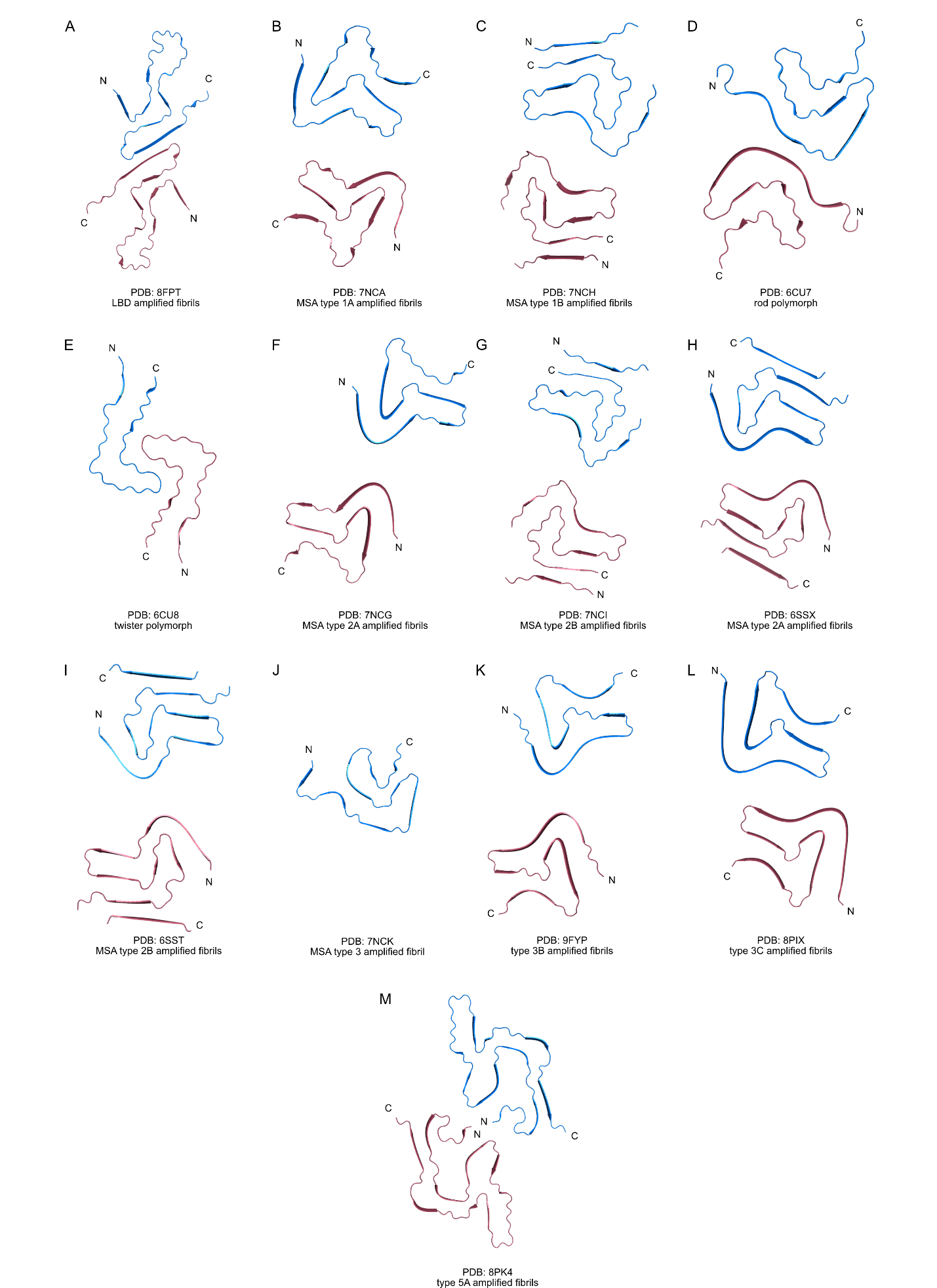


**Suppl. Fig. 1 *In vitro* assembled α-Syn fibrils**

**(A)** *In vitro* α-Syn fibrils adopting Lewy fold, **(B-E)** type 1 *in vitro* MSA filaments formed by two symmetric protofilaments units, **(F-I)** type 2 *in-vitro* MSA filaments formed by two symmetric units, **(J)** *in vitro* MSA single-protofilament, **(G)** rod α-Syn filaments adopting distinct MSA fold, **(K, L)** type 3 *in vitro* MSA filaments formed by two symmetric protofilament units, **(M)** type 5 *in vitro* filaments.


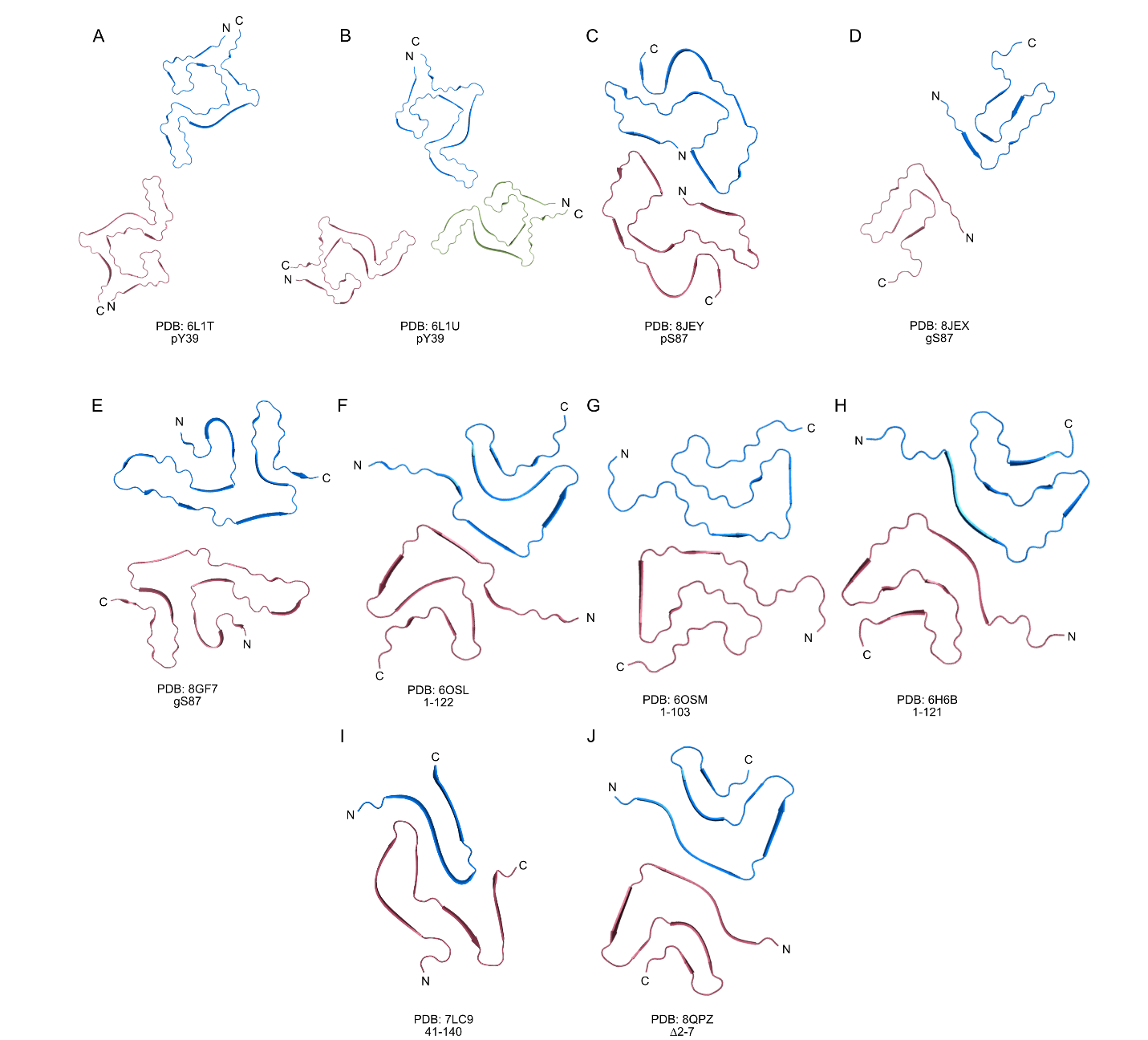


**Suppl. Fig. 2 Structures of α-Syn fibrils with PTMs**

Structures of recombinant α-Syn fibrils carrying PTMs: **(A, B)** pY39, **(C)** pS87, **(D, E)** gS87, **(F)** 1-122, **(G)** 1-103 and **(H)** 1-121 C-terminally truncated α-Syn fibrils, **(I)** 41-140 N-terminally truncated α-Syn fibrils, **(J)** α-Syn fibrils with residues 2-7 deletion.
